# Developing Bayesian EWMA chart for change detection in the shape parameter of Inverse Gaussian process

**DOI:** 10.1371/journal.pone.0301259

**Published:** 2024-05-06

**Authors:** Amara Javed, Tahir Abbas, Nasir Abbas

**Affiliations:** 1 Department of Statistics, Government College University Lahore, Lahore, Pakistan; 2 Department of Mathematics, College of Sciences, University of Sharjah, Sharjah, UAE; 3 Department of Statistics, Government Graduate College, Adhiwal Chowk, Jhang, Pakistan; TU Wien: Technische Universitat Wien, AUSTRIA

## Abstract

Bayesian Control charts are emerging as the most efficient statistical tools for monitoring manufacturing processes and providing effective control over process variability. The Bayesian approach is particularly suitable for addressing parametric uncertainty in the manufacturing industry. In this study, we determine the monitoring threshold for the shape parameter of the Inverse Gaussian distribution (IGD) and design different exponentially-weighted-moving-average (EWMA) control charts based on different loss functions (LFs). The impact of hyperparameters is investigated on Bayes estimates (BEs) and posterior risks (PRs). The performance measures such as average run length (ARL), standard deviation of run length (SDRL), and median of run length (MRL) are employed to evaluate the suggested approach. The designed Bayesian charts are evaluated for different settings of smoothing constant of the EWMA chart, different sample sizes, and pre-specified false alarm rates. The simulative study demonstrates the effectiveness of the suggested Bayesian method-based EWMA charts as compared to the conventional classical setup-based EWMA charts. The proposed techniques of EWMA charts are highly efficient in detecting shifts in the shape parameter and outperform their classical counterpart in detecting faults quickly. The proposed technique is also applied to real-data case studies from the aerospace manufacturing industry. The quality characteristic of interest was selected as the monthly industrial production index of aircraft from January 1980 to December 2022. The real-data-based findings also validate the conclusions based on the simulative results.

## 1 Introduction

A control chart is a vital graphical tool for monitoring and minimizing process variation in statistical process control (SPC). Shewhart [1] initially designed memory-less control chart based on current sample information for process monitoring and used it to identify large process shifts. Later, Roberts [2] designed an EWMA memory-based control chart specifically for small shift detection while giving more weights to the most recent observations. Over the period of time, EWMA is designed for process mean monitoring by [3–5] and for process variation monitoring by [6–8]. Saghir et al. [9] investigated how the estimates affected the geometric-poisson EWMA chart used to monitor the number of defects in the process. EWMA and double-exponentially-weighted-moving-average (DEWMA) were investigated in [10] for the censored data of type-I.

The control charts are designed with respect to the underlying probability of the quality characteristic of interest. Process monitoring relies heavily on the use of waiting time distributions as an important statistical tool. It can be used to analyze and optimize systems that involve waiting times, such as queues, transportation and manufacturing. This helps to improve customer satisfaction, reduce costs, and increase productivity. The applications of waiting time distributions are frequent in the field of manufacturing and industrial engineering. These include serving as a versatile tool for the process analysis and optimization, inventory management, quality control, capacity planning, supply chain management and predictive maintenance. The waiting time distribution has diverse applications in science, such as [11] analyzed and examined the definition, measurement and available data regarding waiting times in healthcare. Waiting time distribution was used by [12] to develop a novel model called the customer-centric two-product split delivery vehicle routing problem for manufacturing system.

In several applications of statistical distributions such as IGD, weibull distribution, and gamma distribution, the shape parameter is a crucial parameter, particularly when it comes to quality control. The monitoring of shape parameter is essential for detecting any changes in the process and ensuring the production of a high-quality product. The literature provides some studies on the monitoring of shape parameter under classical method in manufacturing industry. Such as [13] developed and compared the Shewhart and EWMA charts. The memory-less and memory-type charts were constructed for the weighted power function distribution (WPFD) by [14]. Huberts et al [15] investigated that how well performed the memory-less and memory-type control chart for cautious updating scheme and suggested simple methods. Kinat et al.[16] introduced control charts based on GLM specifically designed for scenarios where the response variable adheres to an IG distribution. Amin et al. [17] constructed Phase II Generalized Linear Models (GLM) based memory type control charts by utilizing deviance residuals and Pearson residuals derived from an IG regression model.

The control charting structures are proposed under classical and Bayesian frameworks with respect to the nature and objective of process evaluation. The Bayesian method is a modern inferential technique which combines the prior information with the sample data to handle uncertainty regarding the parameters of interest. These charts leverage prior knowledge to minimize uncertainty about the process parameters. Unlike the traditional classical control chart, the Bayesian control chart updates the parameters with new data availability and hence providing more precise and timely identification of process errors. The Bayesian EWMA charts are established to handle the parametric uncertainty and to detect small shifts in process parameters [18]. The Bayesian control charting scheme also incorporates various loss functions (LFs) under symmetric and asymmetric scenarios for uncertain process parameters and design different Bayesian EWMA charts such as [19–22]. Aunali and Venkatesan [23] proposed a Bayesian estimator for the quick detection of small shifts in the process mean. Ali [24] introduced Bayesian predictive monitoring of time between events. The Bayesian setup has also been studied in [22,25–35]. Jones et al.[36] reconfigured the CUSUM and EWMA CCs to monitor the process under the Bayesian approach. Seirani et al. [37] presented an economic–statistical design for the Bayesian $\bar{X}$ control chart.

In control charting, the parameters of data distributions are desired to be monitored to assess the shifts occurring in the manufacturing processes. Change / shift in parameters changes the behavior of the process. So, the study of change / shift in parameters is of utmost importance. Therefore, we have attempted to study the process in classical and Bayesian setups of the shape parameter of the waiting time inverse Gaussian distribution. The literature suggested articles on the monitoring of shape parameter of various waiting time distributions that define the manufacturing process. However, there are certain scenarios within the manufacturing industry in which uncertainty of the shape parameter is unavoidable. This study considers the case of parametric uncertainty in manufacturing process and design novel Bayesian EWMA structures for shape parameter when underlying distribution of quality characteristic of interest follows IGD. The Bayesian EWMA charts are based on the Bayes estimates of the posterior distribution of the shape parameter. The posterior distribution is derived after incorporating the prior distribution of the shape parameter with the likelihood function of the sampling distribution of IGD. Then the BEs, PRs and UCLs are derived using various symmetric and asymmetric LFs, such as squared-error loss function (SELF), DeGroot loss function (DLF), modified squared-error loss function (MSELF), K loss function (KLF), weighted squared-error loss function (WSELF) and precautionary loss function (PLF). This study aims to develop Bayesian EWMA control charts using BEs constructed under various LFs and then assess the efficiency of the proposed charts. The new settings of Bayesian EWMA charts are evaluated for possible effect of hyperparameter values, different sample sizes and different values of smoothing constant. As far as the significance of the study related to the real-life data is concerned, we frequently come across the situations when the manufacturing process data follow the waiting time distributions. In such situations, it becomes necessary to monitor the parameters of the distribution. As we have considered the waiting time following the IGD so, we have endeavored to establish the Bayesian EWMA charts based on the shape parameter of the IGD.

The following sections outline the structure of the study: Section 2 covers the Bayesian approach. Section 3 discusses the proposed control charting structures. Performance assessments and elicitation of hyperparameters are explored in Sections 4 and 5 respectively. Section 6 provides a comparative study and a computing algorithm. Numerical study is discussed in Section 7. Section 8 examines the real data application of the method. Section 9 concludes the entire study.

## 2 Bayesian method

Bayesian method effectively handles the parametric uncertainty after using the prior knowledge of the parameters to find the posterior estimates of the unknown process parameters. To determine the posterior distribution of the model parameters given the data, prior knowledge is combined with the current data set. This distribution acts as a foundation for further analysis. This section will give a brief overview of the terminology commonly used in Bayesian methods, derive posterior distribution of the shape parameter of IGD under-study and outline the different loss functions utilized in this context.

### 2.1 Sampling distribution

As elaborated in Section 1, we have used the positively skewed waiting time distribution, i.e., the IGD. Suppose that *x*_1_, *x*_2_, …, *x*_*n*_ denote a random sample of size *n* from the *IGD* (*μ,θ*) Its pdf is as follows:

$$f(x;\mu ,\theta )={\left(\frac{\theta }{2\pi x^{3}}\right)}^{1/2}\exp\left\{-\frac{\theta {\left(x-\mu \right)}^{2}}{2\mu^{2}x}\right\},x>0,\mu >0,\theta >0.$$ (1)

Where μ and *θ* are the location and shape parameters.

### 2.2 Prior and posterior distributions

Bayesian method is utilized to estimate the parameters of a distribution. The concept of a prior distribution refers to the distribution of the parameters. Priors can be divided in to two major groups: informative and uninformative. An informative prior represents prior knowledge about the unknown parameter, and when there is no prior knowledge available, uninformative prior is used. Seirani et al [38] used these types of priors for the Bayesian evaluation. We have assumed gamma prior for the shape parameter *θ*, represented by *p*(*θ*) which is expressed as

$$p(\theta )=\frac{b^{a}}{\Gamma (a)}\theta^{a-1}e^{-b\theta },\theta >0,a>0,b>0.$$ (2)

**Fig 1:**
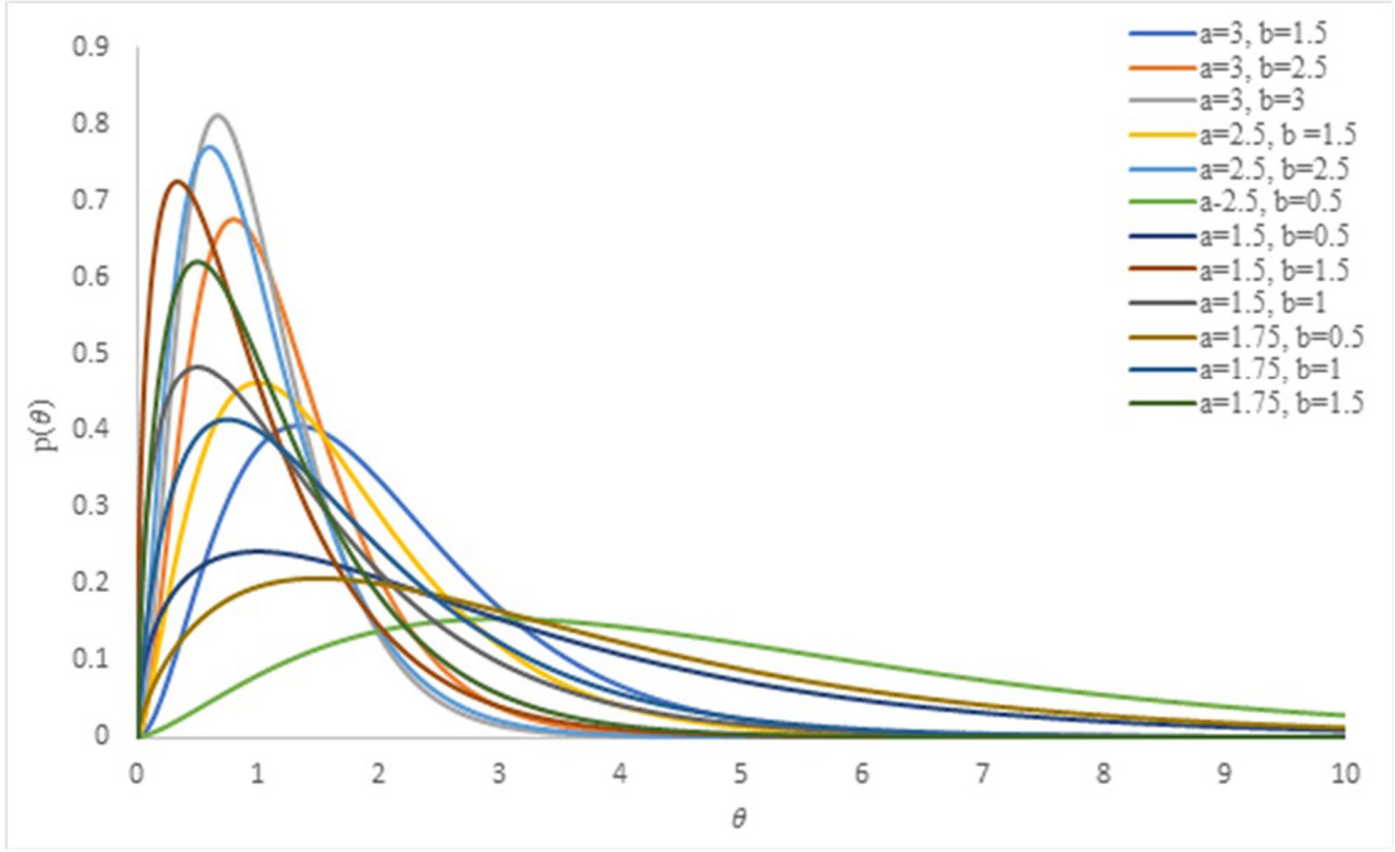
Effect of hyperparameters on the prior distribution.

Where *a* and *b* are the hyperparameters. As we know that a change in the values of the hyperparameters may lead to different nature of the distribution. So, the effect of different combinations of the values of the hyperparameters on the prior gamma is depicted in Fig 1.

The figure exhibits that the prior gamma distribution is of skewed nature in general. However, if the value of hyperparameter *a* is increased and those of *b* are decreased, the shape of the prior distribution becomes symmetric. By combining the prior distribution with sample data, the resulting posterior distribution *p*(*θ*|***x***) is obtained. It includes all information about the unknown parameter and may be expressed as

$$p(\theta |x)=\frac{p(\theta )p(x|\mu ,\theta )}{\int_{\theta }p(\theta )p(x|\mu ,\theta )d\theta }.$$

Which may in proportional form be expressed as

p(θ∣x)∝p(θ)p(x∣μ,θ). (3)

as the divisor defines the total probability and is independent of the parameter of interest *θ*. The likelihood function *p*(***x|****μ,θ*) based on the sampling distribution is expressed as

$$p(x|\mu ,\theta )=\prod_{i=1}^{n}f\left(x_{i};\mu ,\theta \right)={\left(\frac{\theta }{2\pi }\right)}^{n/2}\prod_{i=1}^{n}{\left(\frac{1}{x_{i}^{3}}\right)}^{1/2}\exp\left\{-\frac{\theta }{2\mu^{2}}\sum_{i=1}^{n}\frac{{\left(x_{i}-\mu \right)}^{2}}{x_{i}}\right\}.$$ (4)

So, the posterior distribution of *θ* is defined as

$$p(\theta |x)\propto \frac{b^{a}}{\Gamma (a)}\theta^{a-1}e^{-b\theta }.{\left(\frac{\theta }{2\pi }\right)}^{\frac{n}{2}}\times \prod_{i=1}^{n}{\left(\frac{1}{x_{i}^{3}}\right)}^{\frac{1}{2}}\exp\left\{-\frac{\theta }{2\mu^{2}}\sum_{i=1}^{n}\frac{{\left(x_{i}-\mu \right)}^{2}}{x_{i}}\right\}$$

$$p(\theta |x)\propto \theta^{\frac{n}{2}+a-1}\exp\left\{-\theta \left(b+\sum_{i=1}^{n}\frac{{\left(x_{i}-\mu \right)}^{2}}{2\mu^{2}x_{i}}\right)\right\}.$$ (5)

**Fig 2:**
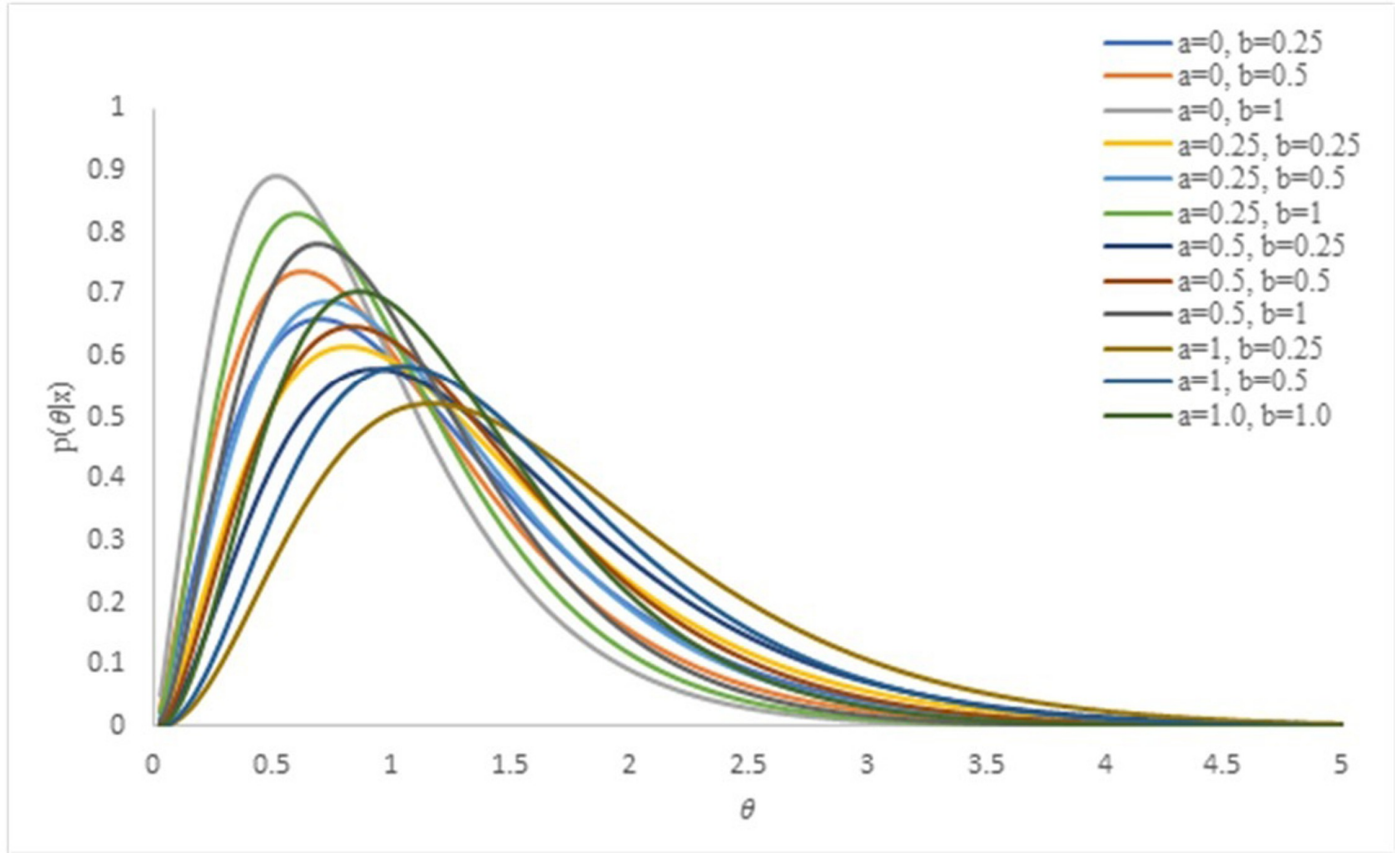
Effect of hyperparameters on posterior distribution.

This means that $\theta |x∼gamma\left(\frac{n}{2}+a,b+\sum_{i=1}^{n}\frac{{\left(x_{i}-\mu \right)}^{2}}{2\mu^{2}x_{i}}\right).$ For simplicity, we denote $\alpha =\frac{n}{2}+a$ and $\beta =b+\sum_{i=1}^{n}\frac{{\left(x_{i}-\mu \right)}^{2}}{2\mu^{2}x_{i}}.$ The effect of hyperparameters on the posterior distribution is visualized in Fig 2.

The posterior distribution as shown in Fig 2, has a skewed shape in general. However, by increasing the values of the hyperparameter *a* and decreasing those for *b*, the shape of the posterior distribution approaches symmetry.

### 2.3 Loss function

Loss functions are fundamental in Bayesian estimation as they provide a principled way to quantify the cost of estimation errors and guide decision-making. The use of loss functions in Bayesian analysis helps ensure that the estimated parameters align with the objectives and consequences of the decision-making process in a given context. A loss function (LF), defined by $L(\theta ,\hat{\theta }),$plays a fundamental role in the Bayesian inference by quantifying the loss or cost involved in estimating the parameter. It minimizes the risk, which is defined as the expected loss and denoted by $E\{L(\theta ,\hat{\theta })\}$ in Bayesian setup. Wald [39] introduced the term LF. In decision theory, the LF is employed to determine the optimal estimator. Various LFs were employed in [19]. There are several loss functions that exist in Bayesian inference such as weighted balanced, Linex, Quadratic, General entropy, Mean absolute error, Mean squared error, Zero one loss functions, etc. This study also includes a variety of LFs that are used for the process monitoring of the shape parameter of IGD and are described in the following subsections.

#### 2.3.1 Squared-error loss function (SELF)

The SELF is a measure that quantifies the accuracy of predictions by squaring the difference between predicted and actual values of the parameter of interest. It highlights larger errors and is widely used in regression analysis, machine learning and control chart evaluation. It is mathematically convenient. The squaring operation ensures a smooth and differentiable function, making it easy to work with in mathematical calculations. In the context of statistics, minimizing the squared differences between observed and predicted values corresponds to maximizing the likelihood under the assumption of normal (Gaussian) distribution of errors. This is often a reasonable assumption in many real-world scenarios. In developing the least square theory, [40,41] suggested the SELF. This LF was used in [33] which is of symmetrical type and is expressed as $L(\theta ,\hat{\theta })={\left(\theta -\hat{\theta }\right)}^{2}$. Here $\hat{\theta }$ denotes the estimator which minimizes the posterior risk. The posterior mean which is represented by ${\hat{\theta }}_{SELF}=E_{SELF}(\theta |x),$ is the BE under SELF.

#### 2.3.2 Weighted SELF (WSELF)

The WSELF is a modified version of SELF. It involves multiplying each prediction error by a weight which allows us to give more importance to specific errors. By adjusting these weights, we can prioritize certain errors over others when evaluating how well a prediction model performs. This asymmetrical LF was used by [42] and expressed as $L(\theta ,\hat{\theta })=\frac{{\left(\theta -\hat{\theta }\right)}^{2}}{\theta },$ where $\hat{\theta }$ denotes an estimator of the unknown parameter *θ*. BE under WSELF is expressed as ${\hat{\theta }}_{WSELF}={\left(E\left(\theta^{-1}|x\right)\right)}^{-1}.$

#### 2.3.3 Modified SELF (MSELF)

The MSELF is a modified version of the SELF that is adjusted or changed to fit the specific needs of a specific problem or situation. These adjustments can involve altering how the squared difference of the predicted and actual values is calculated, adding extra terms or penalties, or using custom weights. This non-symmetrical LF is defined by [43] as ${\left(1-\hat{\theta }/\theta \right)}^{2}$. The BE under MSELF is represented as ${\hat{\theta }}_{MSELF}=\frac{E\left(\theta^{-1}|x\right)}{E\left(\theta^{-2}|x\right)}.$. Here $\hat{\theta }$ is an estimator of the parameter *θ*. The goal of this LF is to create a measurement of prediction accuracy that is customized to handle unique factors.

#### 2.3.4 K Loss Function (KLF)

The KLF is utilized as a cost or loss function in specific applications, especially in machine learning and optimization problems. By minimizing this LF through various optimization techniques, it aids in optimizing model parameters or estimating parameters in statistical models. Its use can contribute to enhancing the performance and accuracy of the models in these domains. This LF was utilized by (cf. [42,44]) and explained as $L(\theta ,\hat{\theta })={\left(\sqrt{\theta /\hat{\theta }}-\sqrt{\hat{\theta }/\theta }\right)}^{2}.$ The representation of BE under KLF is as ${\hat{\theta }}_{KLF}=\sqrt{\frac{E(\theta |x)}{E\left(\theta^{-1}|x\right)}}.$

#### 2.3.5 DeGroot Error Loss Function (DLF)

The DLF is typically defined as ratio of the squared difference between the estimated and true values to the square of the estimated value. The specific form of this LF depends on the problem being addressed. The main purpose of DLF is to evaluate the accuracy of decision making and estimation procedures by assigning penalties for incorrect decisions or the estimates. By minimizing the DLF, the optimal decision rules or estimators can be selected that can lead to improved performance and minimized expected loss. The DLF, $L(\theta ,\hat{\theta })=\frac{{\left(\theta -\hat{\theta }\right)}^{2}}{{\hat{\theta }}^{2}},$ was introduced by [45] and was later utilized by [46] to obtain BEs. The estimator $\hat{\theta }$, which minimizes the PR, is used for parameter *θ*. The BE for DLF can be calculated as ${\hat{\theta }}_{DLF}=\frac{E\left(\theta^{2}|x\right)}{E(\theta |x)}.$

#### 2.3.6 Precautionary Loss Function (PLF)

Loss functions like the precautionary loss function are designed to create conservative estimators that prioritize avoiding underestimation. These estimators are particularly valuable in situations where underestimation could lead to severe consequences. The PLF is a straightforward and valuable asymmetric LF that helps to address this concern. This LF is presented by [47,48]. It is frequently used in the fields of risk management, decision making and financial planning. PLF is represented as $L(\theta ,\hat{\theta })=\frac{{\left(\theta -\hat{\theta }\right)}^{2}}{\hat{\theta }}$ and its BE under PLF is given as ${\hat{\theta }}_{PLF}=\sqrt{E\left(\theta^{2}|x\right)}.$

This study evaluates the performance of these LFs in terms of BEs and PRs. Table 1 provides a summary of the LFs under investigation along with their expressions, associated BEs and PRs. The findings of this study will contribute to improve the efficiency of different loss functions in the Bayesian framework.

**Table 1:** Bayes estimators and posterior risks under different loss functions.

Loss function	Mathematical expression	Bayes estimator (BE)	Posterior risk (PR)
**SELF**	${\left(\theta -\hat{\theta }\right)}^{2}$	$E(\theta |x)=\frac{\alpha }{\beta }$	$E\left(\theta^{2}|x\right)-{\left(E(\theta |x)\right)}^{2}=\frac{\alpha }{\beta^{2}}$
**WSELF**	$\frac{{\left(\theta -\hat{\theta }\right)}^{2}}{\theta }$	${\left(E\left(\theta^{-1}|x\right)\right)}^{-1}=\frac{\alpha -1}{\beta }$	$E(\theta |x)-{\left(E\left(\theta^{-1}|x\right)\right)}^{-1}=\frac{1}{\beta }$
**MSELF**	${\left(1-\hat{\theta }|\theta \right)}^{2}$	$\frac{E\left(\theta^{-1}|x\right)}{E\left(\theta^{-2}|x\right)}=\frac{\alpha -2}{\beta }$	$\frac{1-{\left[E\left(\theta^{-1}|x\right)\right]}^{2}}{E\left(\theta^{-2}|x\right)}=\frac{1}{\left(\alpha -1\right)}$
**KLF**	${\left(\sqrt{\theta |\hat{\theta }}-\sqrt{\hat{\theta }|\theta }\right)}^{2}$	$\sqrt{\frac{E(\theta |x)}{E\left(\theta^{-1}|x\right)}}=\frac{\sqrt{\alpha (\alpha -1)}}{\beta }$	$\begin{array}{c} 2\left[\sqrt{E(\theta |x)E\left(\theta^{-1}|x\right)}-1\right]\\ =2\left[\sqrt{\left(\frac{\alpha }{\alpha -1}\right)}-1\right] \end{array}$
**DLF**	$\frac{{\left(\theta -\hat{\theta }\right)}^{2}}{d^{2}}$	$\frac{E\left(\theta^{2}|x\right)}{E(\theta |x)}=\frac{\alpha +1}{\beta }$	$\frac{var(\theta |x)}{E\left(\theta^{2}|x\right)}=\frac{1}{\alpha +1}$
**PLF**	$\frac{{\left(\theta -\hat{\theta }\right)}^{2}}{\hat{\theta }}$	$\sqrt{E\left(\theta^{2}|x\right)}=\frac{\sqrt{\alpha (\alpha +1)}}{\beta }$	$\begin{array}{c} 2\left(\sqrt{E\left(\theta^{2}|x\right)}-E(\theta |x)\right)\\ =2\left[\frac{\sqrt{\alpha (\alpha +1)}-\alpha }{\beta }\right]\alpha \end{array}$

## 3 The proposed control charting structures

This section presents the detailed structures of the Bayesian and classical EWMA control chart of the shape parameter of IGD. The EWMA control chart exhibit higher sensitivity in detecting small process shifts as compared to the Shewhart type control chart [49]. It is important to note that in numerous practical applications, there is a practical interest in detecting one-sided changes, which can be either increases or decreases as reported in [50]. For example, in certain situations, it is crucial to ensure that leakage remains below a specific threshold, while breaking strength must meet or exceed a fixed nominal value. As the sampling and the posterior distribution of the shape parameter are positively skewed, so in this study, we have constructed only the UCLs under different settings of BEs and PRs. The following subsections first describe the EWMA charting structure for the shape parameter under classical setup and then the Bayesian EWMA charts based on different LFs.

### 3.1 The classical EWMA control chart

Let *x*_1_, *x*_2_, …, *x*_*n*_ form a random sample from IGD, i.e., *X*~*IGD*(*μ, θ*) where *θ* is the unknown shape parameter. For the *i*^th^ sample, the estimated statistic ${\hat{\theta }}_{i}$ of the shape parameter *θ* is used to obtain the EWMA statistic *z*_*i*_ under classical setup and is given as

$$z_{i}=\lambda {\hat{\theta }}_{i}+(1-\lambda )z_{i-1};i=1,2,3,\ldots ,n\;\text{and}\;z_{0}=\theta_{0},$$ (6)

where *λ* stands for the smoothing constant, and *z*_0_ = *θ*_0_ is the value of the shape parameter in the in-control process. The classical estimator of *θ* along with its mean and variance are given as

$$\hat{\theta }=\frac{n}{\sum_{i=1}^{n}x_{i}^{-1}-n{\bar{x}}^{-1}},$$ (7)

$$E(\hat{\theta })=E\left[\frac{n}{\sum_{i=1}^{n}x_{i}^{-1}-n{\bar{x}}^{-1}}\right],$$ (8)

$$var(\hat{\theta })=2{\left[\sum_{i=1}^{n}x_{i}^{-1}-n{\bar{x}}^{-1}\right]}^{-2}.$$ (9)

The UCL can be constructed as

$$UCL=E(\hat{\theta })+L\sqrt{var(\hat{\theta })\left(\frac{\lambda }{2-\lambda }\right)\left[1-{\left(1-\lambda \right)}^{2i}\right]}.$$ (10)

### 3.2 The Bayesian EWMA control chart

This subsection describes the Bayesian EWMA control chart based on the BEs of the shape parameter under the LFs under study. The Bayesian EWMA plotting statistic under any LF is defined as

$$z_{i}=\lambda {\hat{\theta }}_{LF(i)}+(1-\lambda )z_{i-1};i=1,2,3,\ldots ,n\;\text{and}\;z_{0}=\theta_{0}.$$ (11)

Where ${\hat{\theta }}_{LF(i)}$ denotes the BE of the shape parameter for any specific loss function and the i^th^ sample, and rest of the notations are defined earlier. The BE under SELF, its expected value, PR, and the corresponding UCL based on the Bayesian EWMA chart is designed as follows:

$${\hat{\theta }}_{SELF}=\frac{\alpha }{\beta }=\frac{\frac{n}{2}+a}{b+\sum_{i=1}^{n}\frac{{\left(x_{i}-\mu \right)}^{2}}{2\mu^{2}x_{i}}}$$ (12)

The mean and variance of the estimator *θ* are derived as:

$$E{\left(\hat{\theta }|x\right)}_{SELF}=E\left(\frac{\alpha }{\beta }\right)=E\left(\frac{\frac{n}{2}+a}{b+\sum_{i=1}^{n}\frac{{\left(x_{i}-\mu \right)}^{2}}{2\mu^{2}x_{i}}}\right)$$ (13)

$$var{\left(\hat{\theta }|x\right)}_{SELF}=var\left(\frac{\alpha }{\beta }\right)=var\left(\frac{\frac{n}{2}+a}{b+\sum_{i=1}^{n}\frac{{\left(x_{i}-\mu \right)}^{2}}{2\mu^{2}x_{i}}}\right)$$ (14)

The similar quantities based on the other LFs under study are derived and reported in Table 1. The general form of UCL of Bayesian EWMA for any specific LF is

$$UCL_{LF}=\text{E}{\left(\hat{\theta }|x\right)}_{LF}+L\sqrt{var{\left(\hat{\theta }|x\right)}_{LF}\left(\frac{\lambda }{2-\lambda }\right)\left[1-{\left(1-\lambda \right)}^{2i}\right]}.$$ (15)

And the corresponding UCL of Bayesian EWMA chart under SELF is defined as

$$UCL_{SELF}=\text{E}{\left(\hat{\theta }|x\right)}_{SELF}+L\sqrt{var{\left(\hat{\theta }|x\right)}_{SELF}\left(\frac{\lambda }{2-\lambda }\right)\left[1-{\left(1-\lambda \right)}^{2i}\right]}$$ (16)

The UCLs for the Bayesian EWMA chart based on all the other LFs can similarly be evaluated using the information contained in Table 1.

## 4 Performance measures

The control charting structures under classical and Bayesian frameworks designed in Section 3 are further assessed using different performance measures, such as ARL, SDRL and MRL. These measures are well-known for evaluating the efficiency of a control chart at a certain shift value. The ARL values are widely employed performance measures for the evaluation of the control chart [50,51]. It shows how many samples are usually taken before a signal is detected. A lower ARL suggests that process changes are detected more quickly, whereas a greater ARL indicates a slower response (cf. [52–55]).

The SDRL is another statistical performance measure which quantifies the dispersion of the lengths of runs in a set of data. This technique is frequently used to assess a system’s consistency and stability over time and provides information on the reliability of run length behavior. The MRL is an essential measure used to assess individual control charts and determine the level of skewness in the run length (RL) performance. The MRL value is obtained by selecting the *((m+1)/2)*^*th*^ value from the ordered RLs. In addition to ARL values, the SDRL (for spread) and MRL (for skewness) are also computed as supplemental indicators. The control chart that yields smaller ARL, SDRL, and MRL values is the most effective chart (cf. [56–59]).

## 5 Elicitation of hyperparameters values

**Fig 3:**
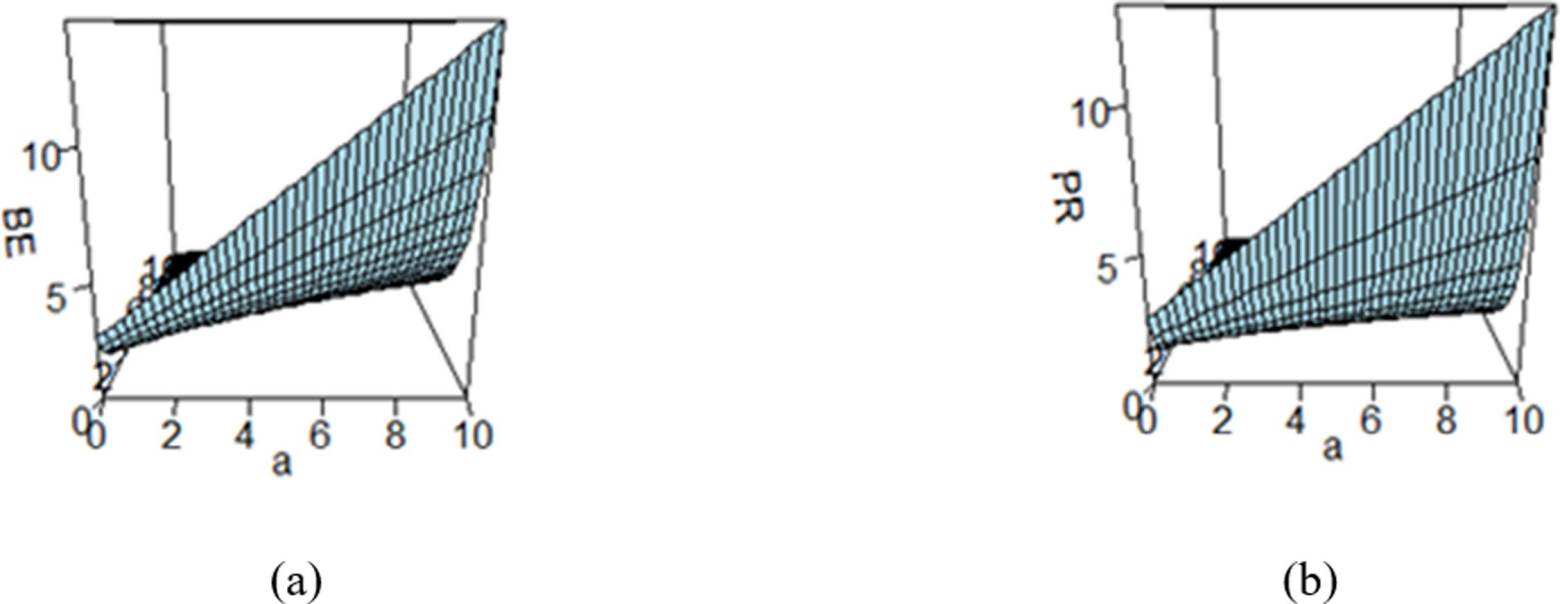
Effect of hyperparameters on the (a) BE and (b) PR under SELF.

**Fig 4:**
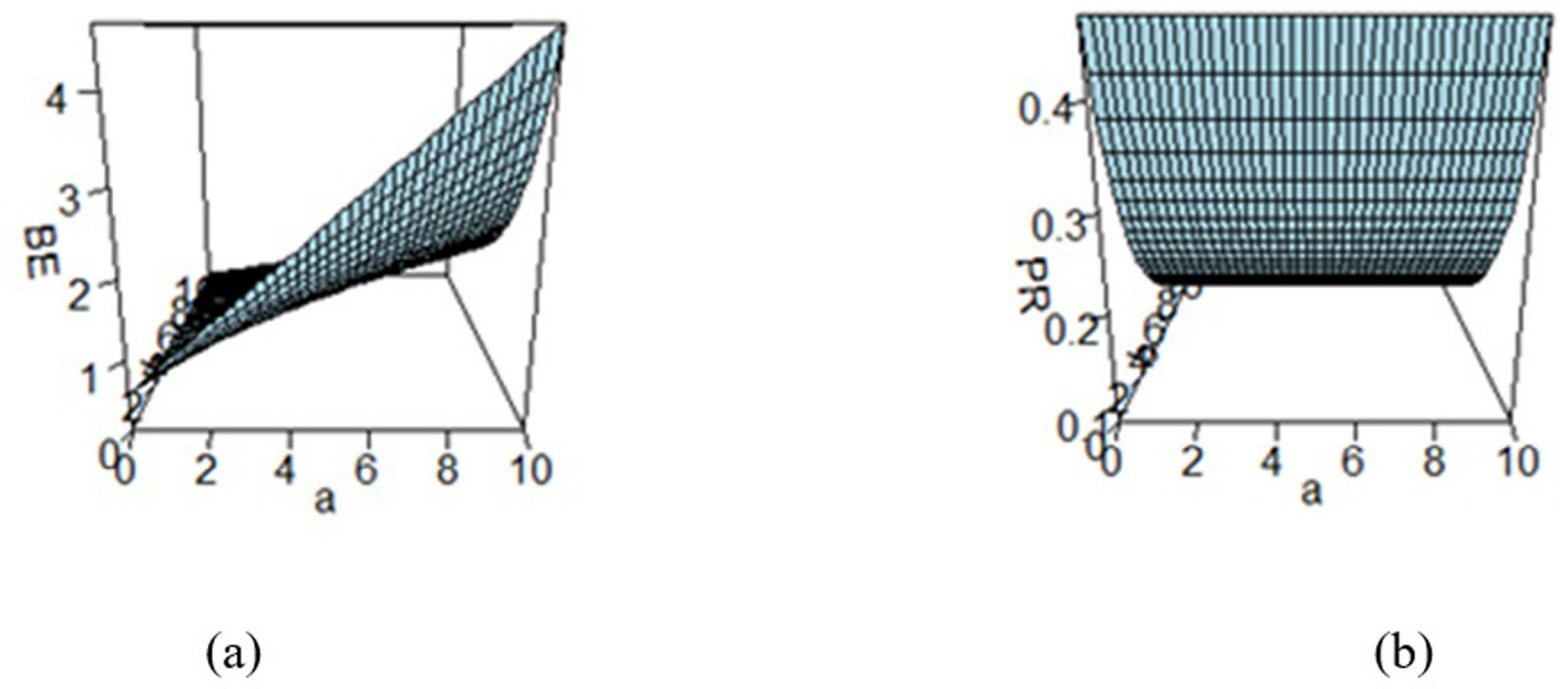
Effect of hyperparameters on the (a) BE and (b) PR under WSELF.

**Fig 5:**
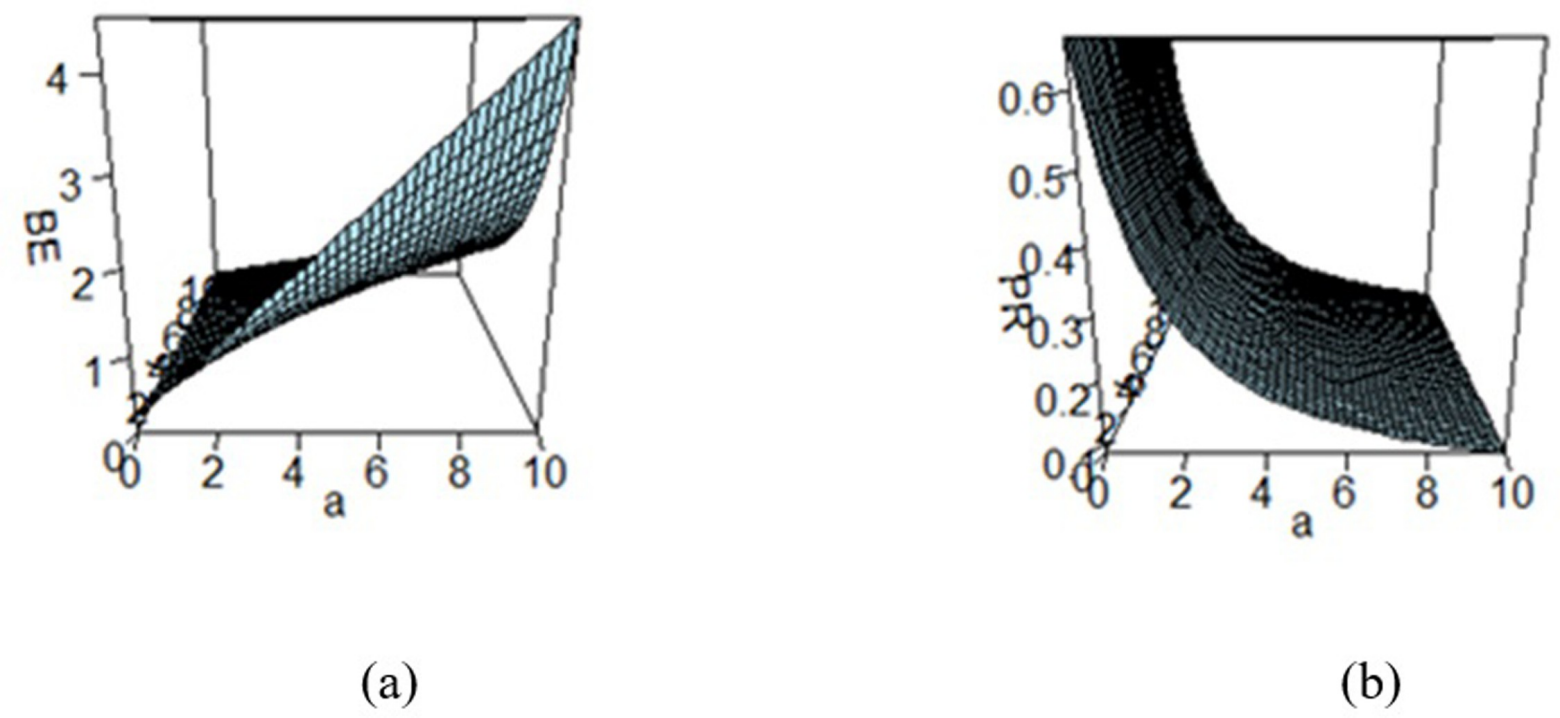
Effect of hyperparameters on the (a) BE and (b) PR under MSELF.

**Fig 6:**
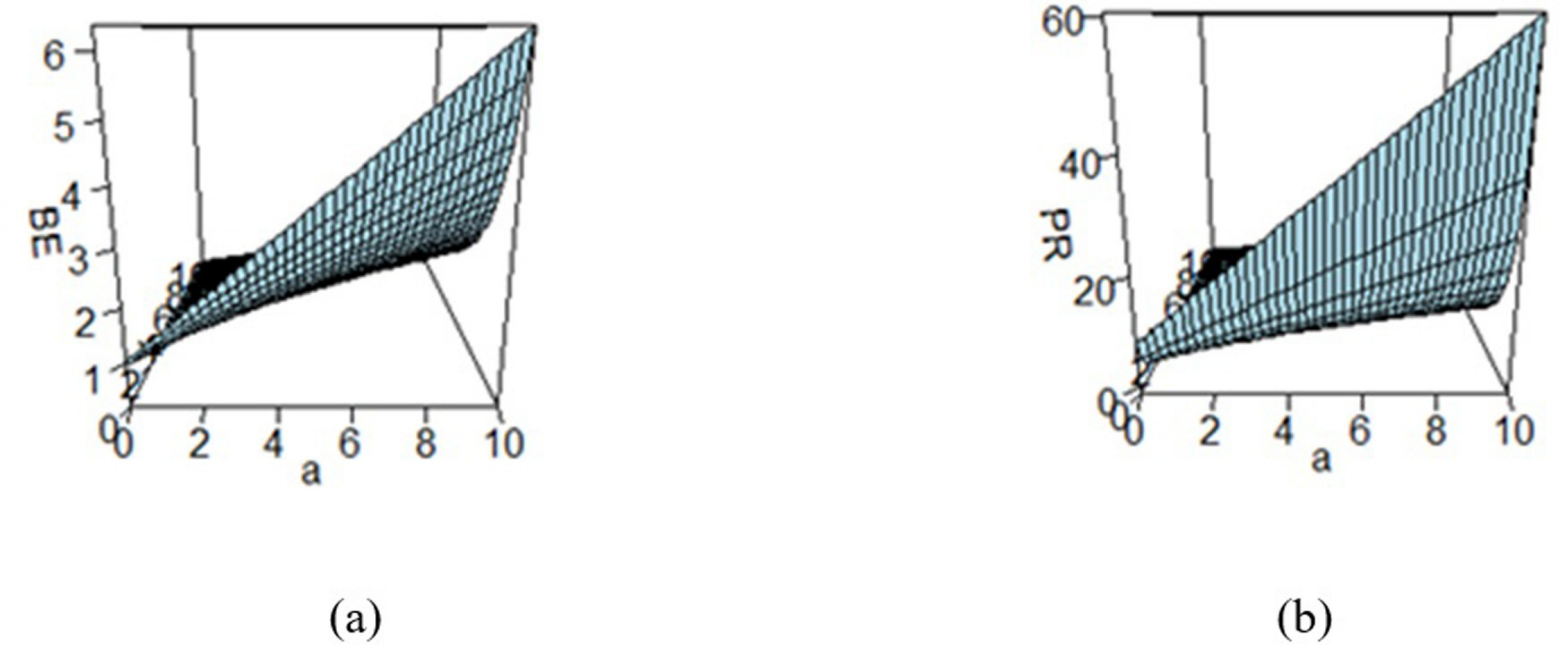
Effect of hyperparameters on the (a) BE and (b) PR under KLF.

**Fig 7:**
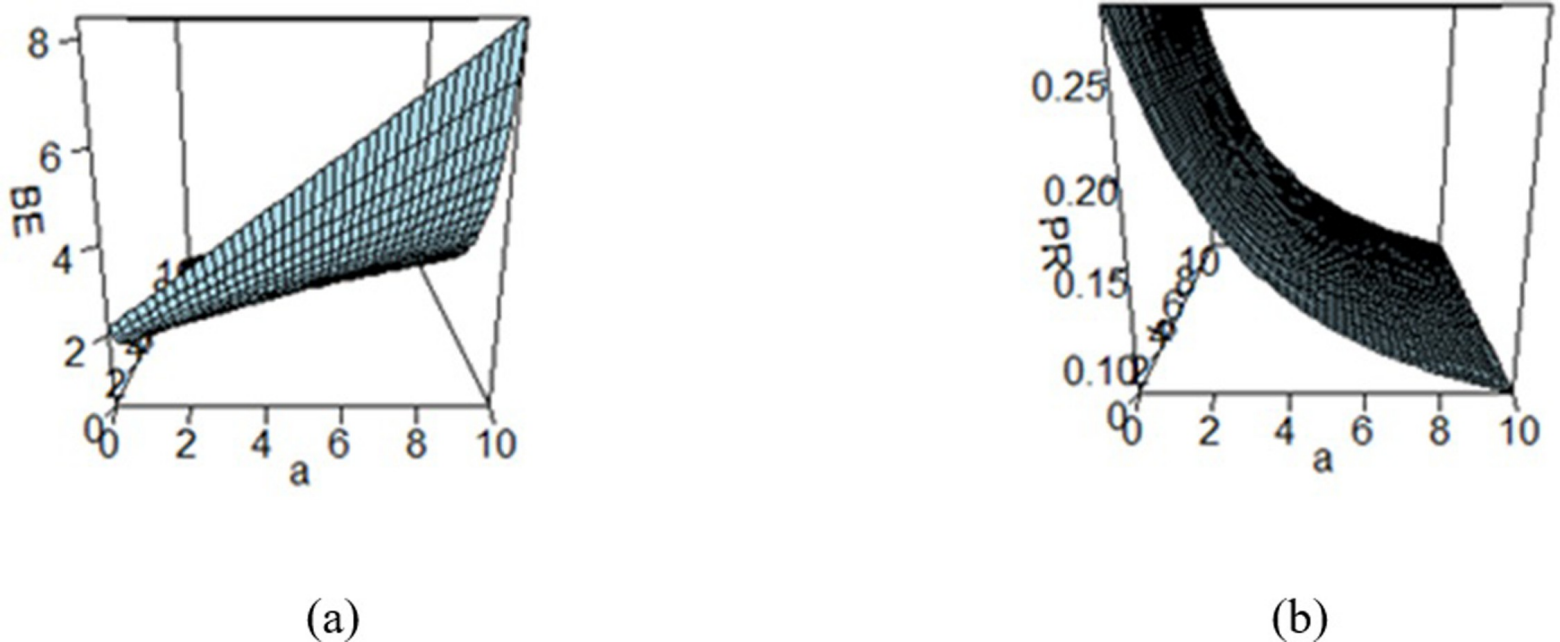
Effect of hyperparameters on the (a) BE and (b) PR under DLF.

**Fig 8:**
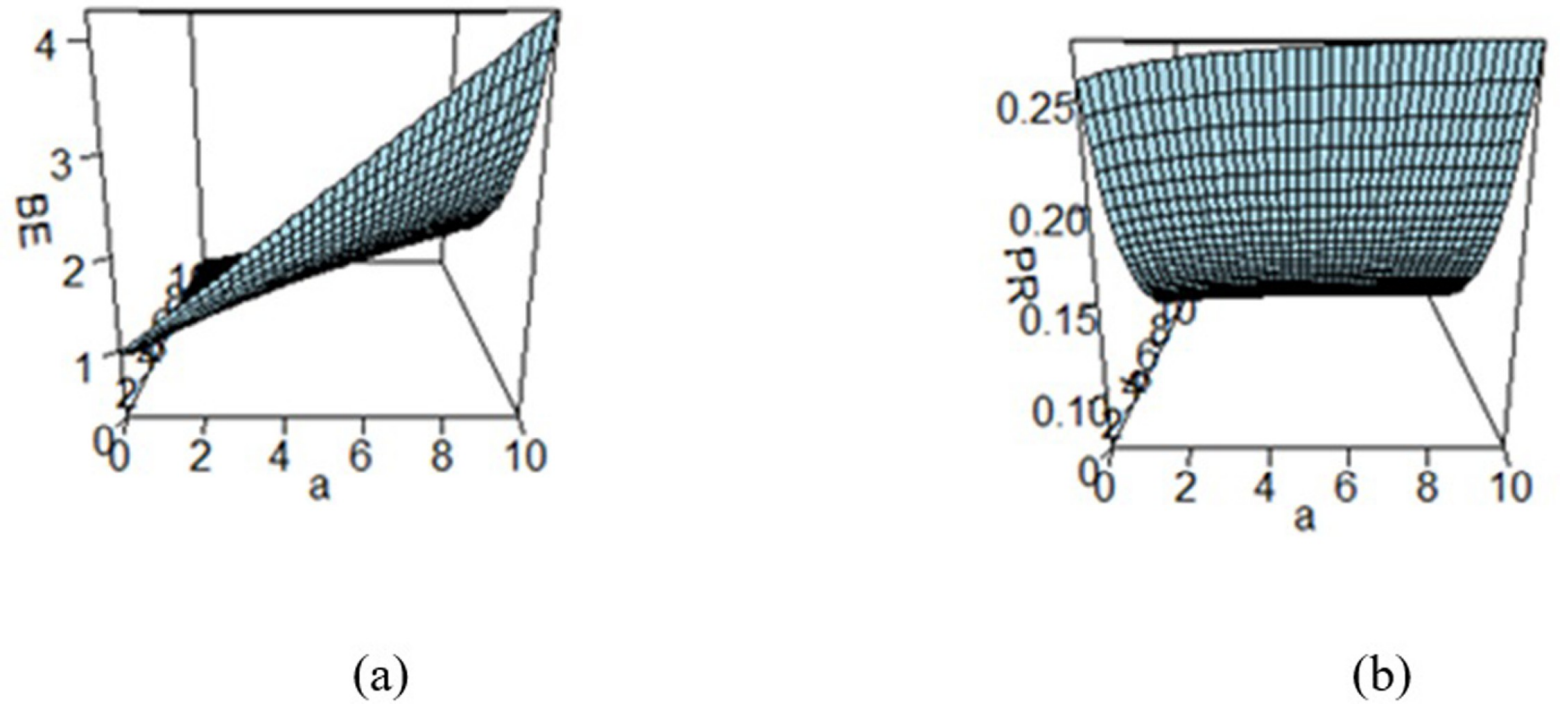
Effect of hyperparameters on the (a) BE and (b) PR under PLF.

Bayes estimates are significantly dependent on the prior distribution which is further dependent on the hyperparameters. So, it is not out of the context to say that the Bayes estimates also depend on the hyperparameters. It is the earnest need of applying Bayesian approach to elicit the values of the hyperparameters in accordance with the experts’ opinions. When a large amount of information about the parameter(s) is available, it is important to quantify this knowledge in the form of a prior and subsequently utilize it in the Bayesian analysis. Elicitation involves the precise measurement of prior information through a systematic process and various elicitation techniques were developed by (cf. [60–66]). To see the impact of the hyper parametric values on the BEs and PRs, these quantities are evaluated across a range of hyperparameters *a* and *b*, and the resulting estimates are displayed in figures (cf. Figs 3–8). These figures reflect the effect of the values of hyperparameters a and b varying from 0 to 10 on Bayes estimates and posterior risk under different loss functions under study. The values of the hyperparameters a and b vary from 0 to 10.

Figs 3–8 are three-dimensional figures plotted among *a*, *b*, and BEs (or PRs) with values of all of these quantities reported on three axes of the figures. These present a study on the impact of change in the hyperparameters *a*, *b* on BEs and PRs using the loss functions under study. The values of hyperparameters vary from zero to infinity. In the study, it is found that as the hyperparameter *a* increases and *b* decreases, the values of BE increase across all the LFs. For SELF, WSELF, KLF, and PLF, the values of PR increase as the hyperparameter *a* increases and *b* decreases and the vice versa. But for any fixed value of *b* for MSELF and DLF, the PR values decrease by increasing the values of *a* and the vice versa. These results highlight the importance of selecting appropriate hyperparameters for a given loss function to optimize the Bayesian EWMA chart performance.

In cases where there is insufficient information about the hyperparameters, it is common to adopt a mild information approach, as proposed by [50]. By following [50], we have used the values of *a* = 3.0 and *b* = 0.1466 for the hyperparameters of the gamma prior. These values will be used throughout the upcoming Bayesian analysis in this study.

## 6 Comparative study

This section presents a comparative analysis of control chart for monitoring the shape parameter using both classical and Bayesian approaches. The study aims to assess the effectiveness of various Bayesian charts and compare them to their classical counterparts. To conduct the analysis, many researchers utilized the Monte Carlo simulation method and developed an algorithm. Shift values are incorporated into the shape parameter and the resulting measures of run lengths including ARL, SDRL, and MRL values are evaluated. These values are employed to assess the efficiency of the suggested control chart. The control chart is supposed to be the best if the value of ARL_1_ for a particular shift is small. The values of ARL_0_ have been fixed by adjusting the values of L under various LFs. The ARL_0_ is specified at 370. Performance of the designed chart is assessed at *λ* = 0.1 and 0.2. The following algorithm is proposed to monitor the process using Bayesian method with different loss functions

**Step 1:** Establishing the control limits
  1. Fix the value of L and *λ* and estimate the control limits.
  2. Generate random sample from IGD for the in-control process.
  3. For the specified loss functions, compute the EWMA statistic *Z*_*i*_ for the classical and Bayesian control chart.
  4. Repeat steps 1–3 if the plotted statistic falls within the control limits.
  5. When the plotted statistic falls outside the limits, record the number of generated samples.
  6. Compute *ARL*_1_ after repeating the steps 3–5 for 100000 times.
**Step 2:** Examine the ARL that is out-of-control.
  1. Generate the random sample from the IGD for the shifted process.
  2. Compute the EWMA statistic *Z*_*i*_.
  3. If plotting statistic is within the specified limits, proceed to repeat steps 1–2. Otherwise, make a note of the number of generated points.
  4. To obtain the *ARL*_1_, *SDRL*_1_ and *MDRL*_1_ values, the steps (1–3) are repeated 50,000 times.

In this study, we calculated the *ARL*, *SDRL*, *and MRL* using both frequentist and Bayesian setups with various loss functions for sample sizes of *n* = 5, 10, 15. The *ARL* values were determined for in-control situations with *ARL*_0_ set at 370 and for shifts of different magnitudes ranging from 1.2–3.0 (i.e., *δ = 1*.*2*, *1*.*4*, *1*.*6*, *1*.*8*, *2*.*0*, *2*.*2*, *2*.*6*, *3*.*0*). Furthermore, *ARL*_1_, *SDRL*_1_, and *MRL*_1_ values are computed for each shift.

**Fig 9:**
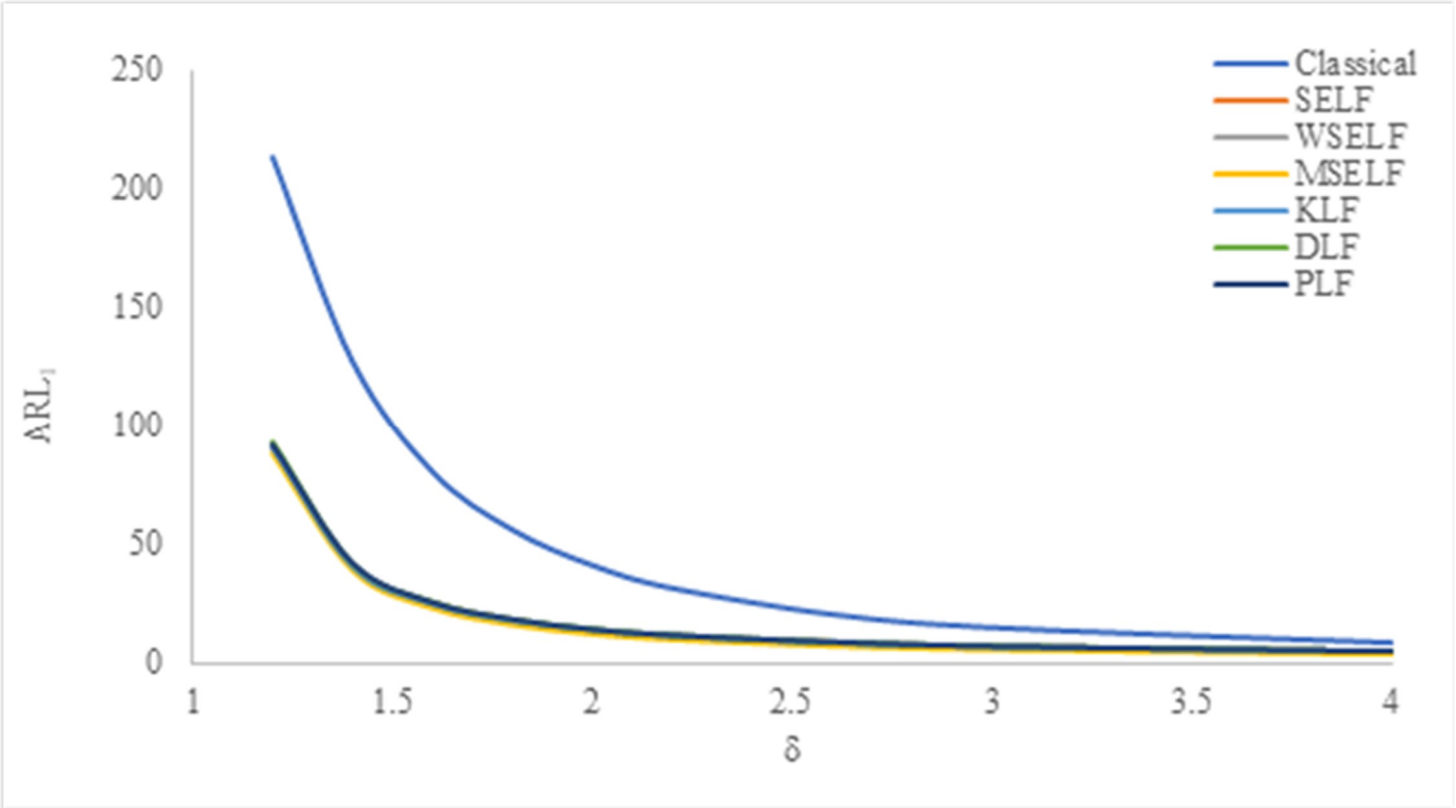
ARL_1_ under classical and Bayesian setups for n = 5 at *λ* = 0.1.

**Fig 10:**
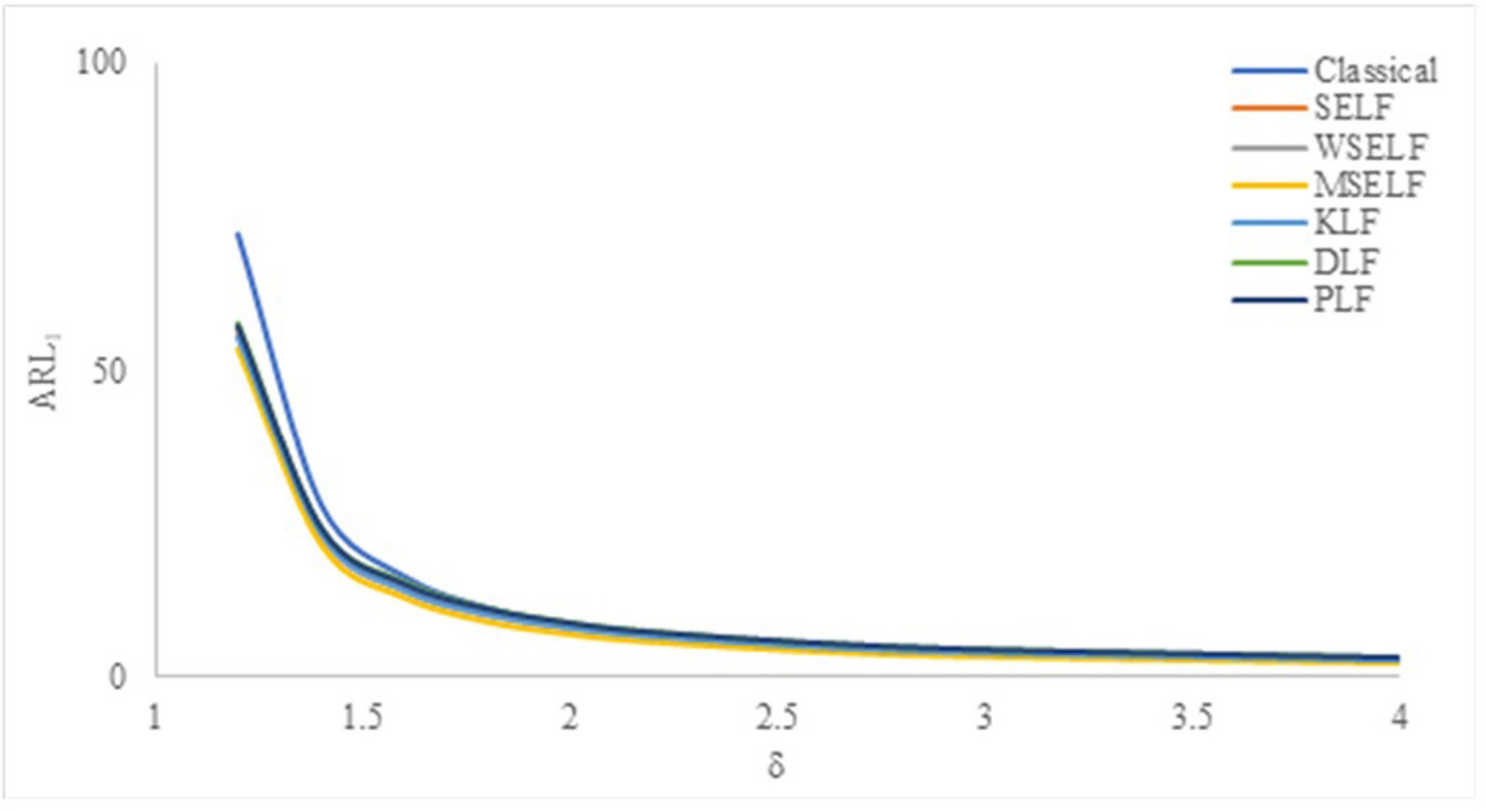
ARL_1_ under Classical and Bayesian setups for n = 10 at *λ* = 0.1.

**Fig 11:**
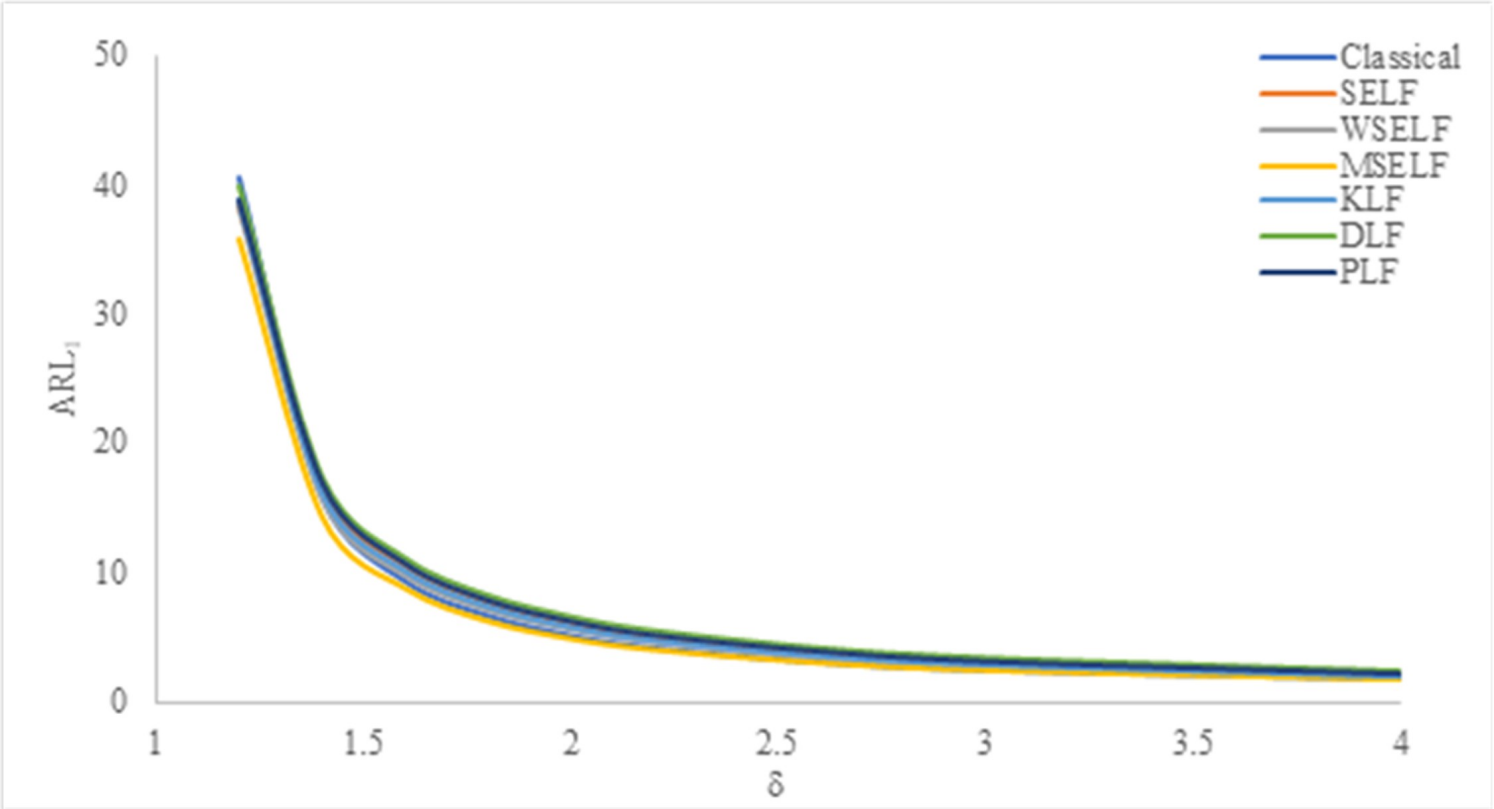
ARL_1_ under classical and Bayesian setups for n = 15 at *λ* = 0.1.

**Fig 12:**
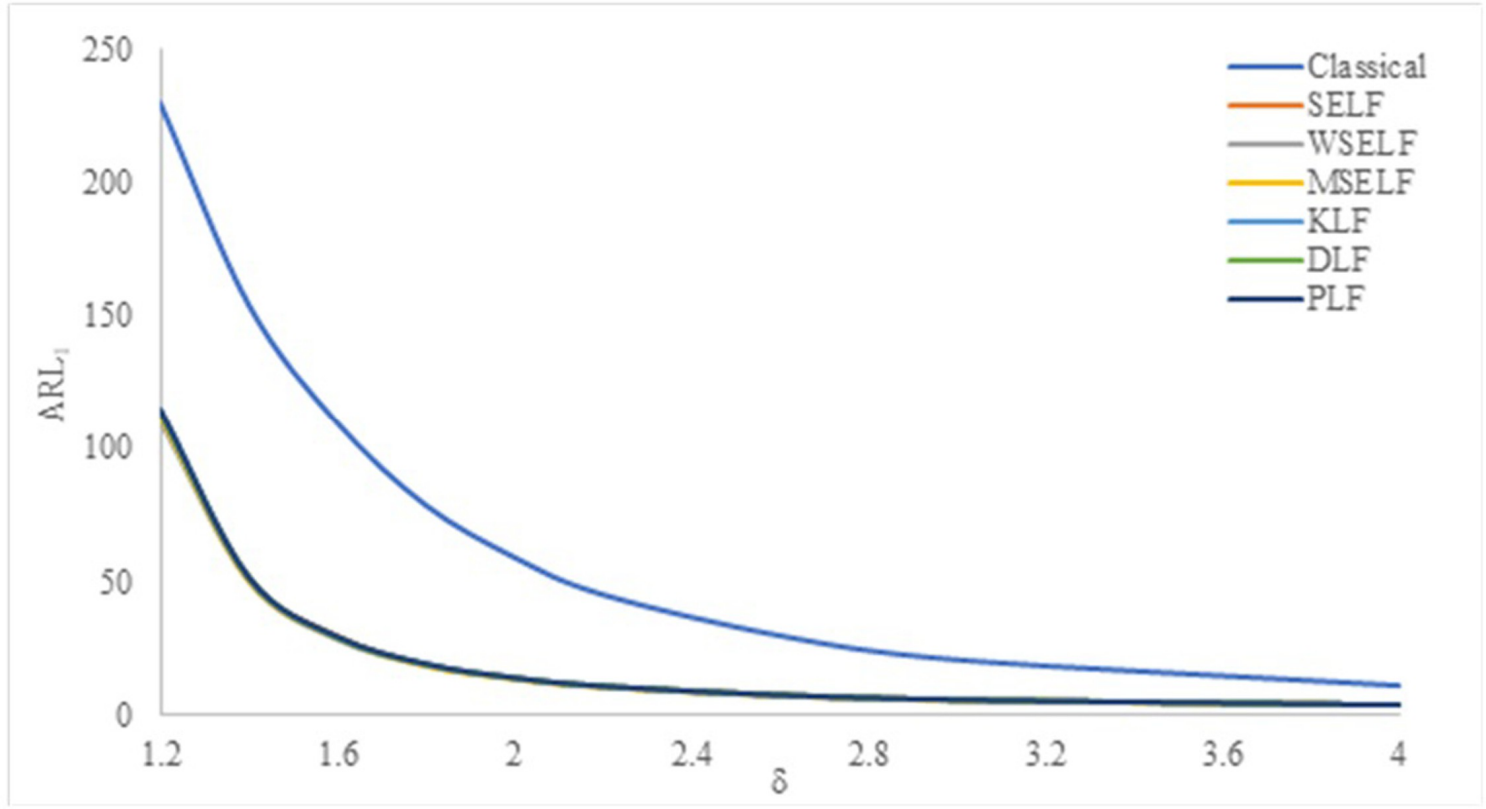
ARL_1_ under classical and Bayesian setups for n = 5 at *λ* = 0.2.

**Fig 13:**
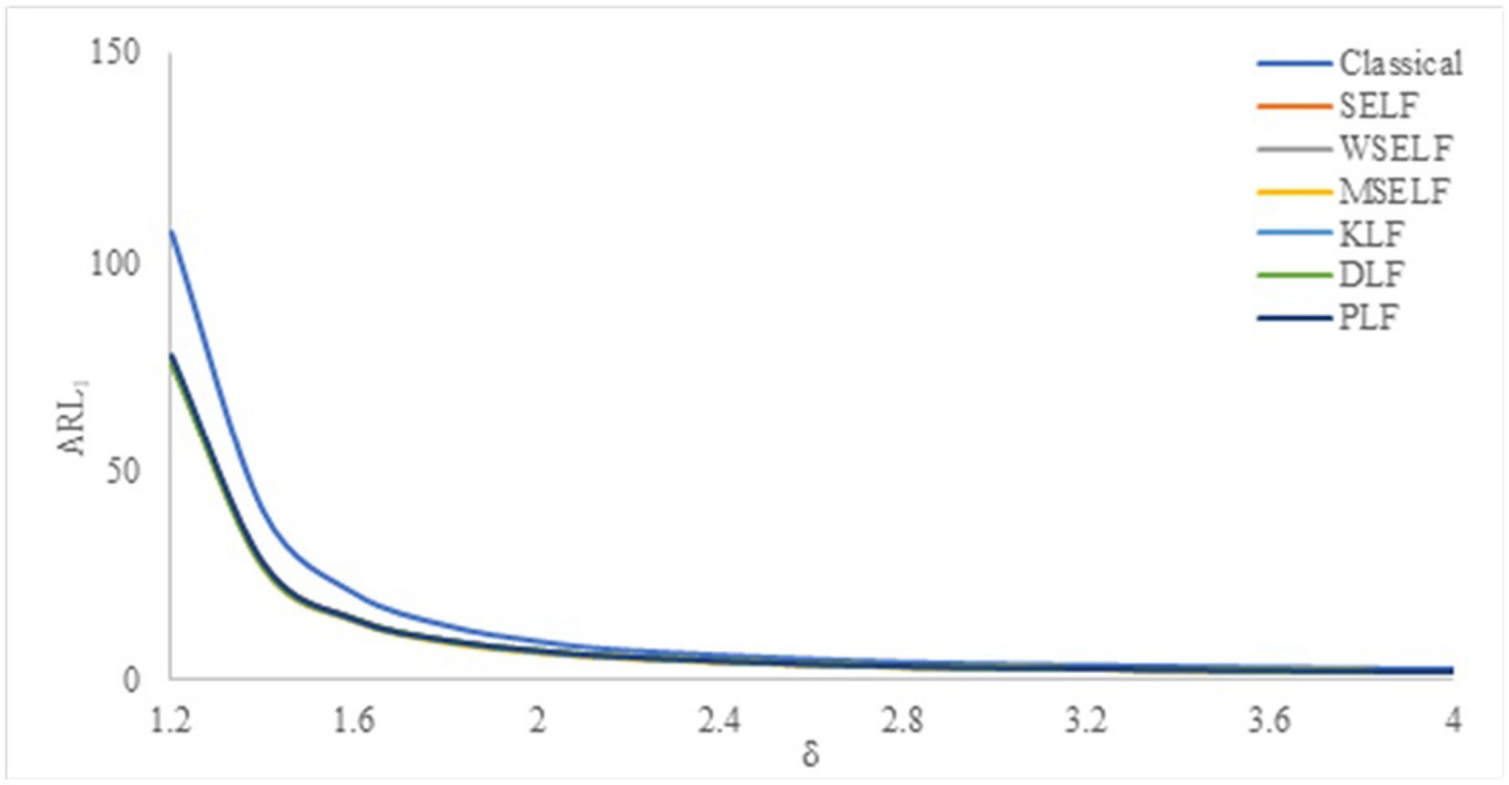
ARL_1_ under classical and Bayesian setups for n = 10 at *λ* = 0.2.

**Fig 14:**
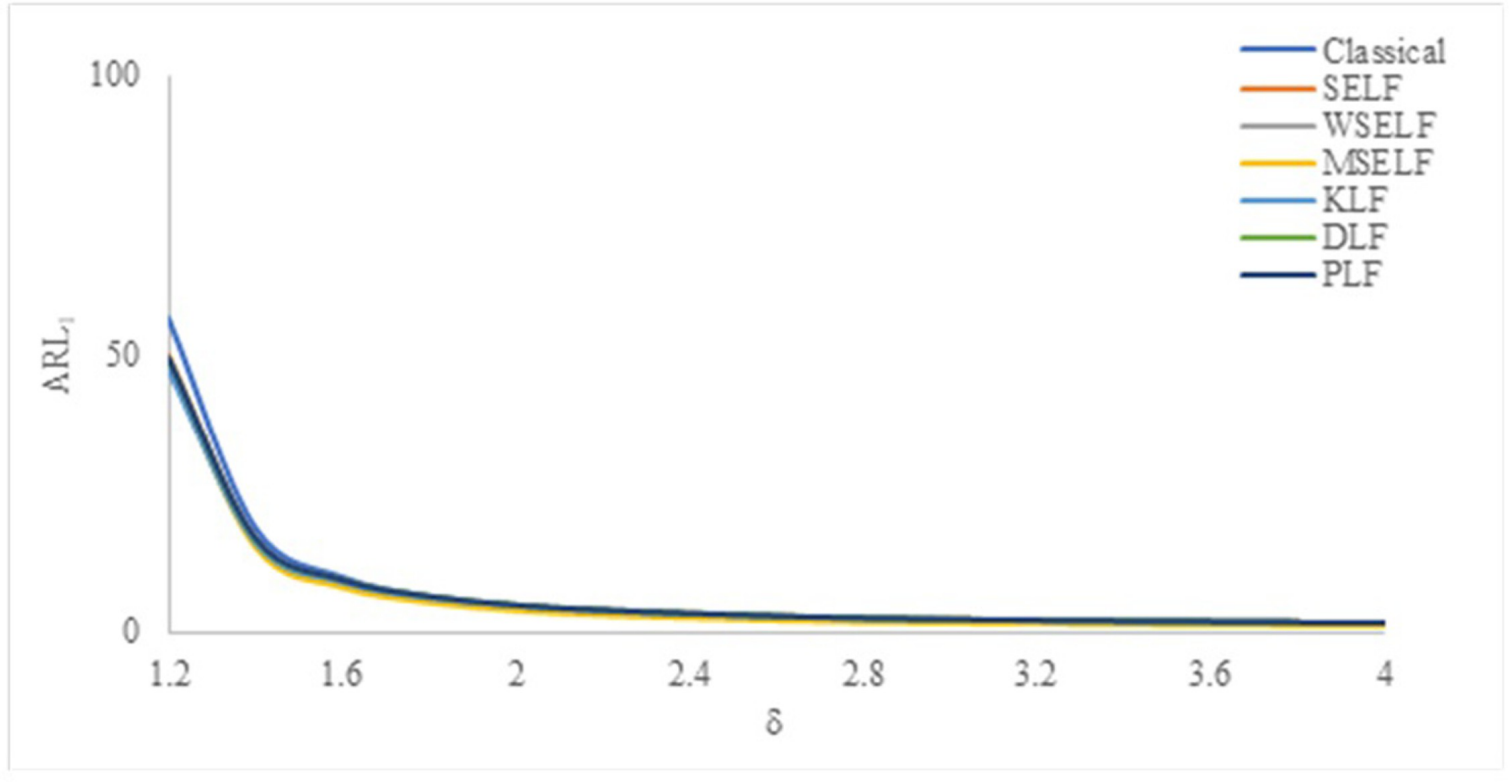
ARL_1_ under classical and Bayesian setups for n = 15 at *λ* = 0.

The results were summarized in Tables 2–7 and visually presented in Figs 9–14. When the shape parameter *(θ)* deviates from its in-control state, the manufacturing process is considered unstable. In the in-control state where *δ = 1*, it signifies the absence of any shift in the shape parameter, indicating a stable state of the process while *δ>1* represents an increase in the process shape parameter. A comprehensive simulation study is conducted to examine the performance of control chart designed with different loss functions in comparison to classical control chart. The results presented in Tables 2–7 indicates that the control charts designed using different loss functions outperformed their classical counterparts. The simulation study yielded several key findings, which are summarized below at *λ* = 0.1 and 0.2.

1. In this study, it was observed that the *ARL*_1_ values tend to decrease as the shift *(δ)* values increase. As an example, when *n = 5* and *δ = 1*.*2*, *ARL*_1_ was found to be *212*.*97*, whereas at *δ = 1*.*4*, the *ARL*_1_ decreased to *127*.*29* at *L = 2*.*26* under classical setup (cf. Table 2).
2. The *ARL*_1_ values were found to exhibit noteworthy improvement for various shift *(δ)* values and control chart under various LFs. For example, when *n = 5*, *δ = 1*.*2*, *ARL*_1_ *= 90*.*76* at *L = 3*.*13* whereas at *δ = 1*.*4* under SELF, the *ARL*_1_ value decreased to *40*.*37* (cf. Table 2).
3. When sample sizes are increased, the *ARL*_1_ values decrease. For instance, for *n = 5* and *δ = 1*.*2*, the *ARL*_1_ value is *91*.*46* at *L = 3*.*13*, whereas for *n = 10* and *δ = 1*.*2*, the *ARL*_1_ value is *55*.*62* at *L = 2*.*95* for WSELF. (cf. Tables 2 and 3). This indicates that increasing the sample size can lead to improved performance in terms of *ARL*_1_ values.
4. *ARL*_1_ values for different loss functions also exhibit improvement. For example, with *δ = 1*.*2* and *n = 5*, the *ARL*_1_ values are *90*.*76* for SELF, *91*.*46* for WSELF, *88*.*29* for MSELF, *91*.*11* for KLF, *93*.*01* for DLF, and *91*.*89* for PLF. Interestingly, all the loss functions exhibit the same value of *L = 3*.*13*, indicating that they perform similarly in this regard. (cf. Table 2).
5. At large sample sizes *(n)*, the control chart using the MSELF loss function outperforms than other loss functions. For instance, with *δ = 1*.*2* and *n = 15*, the *ARL*_1_ value for SELF is *38*.*28* at *L = 2*.*83*, for WSELF it is *38*.*15* at *L = 2*.*85*, for MSELF it is *35*.*88* at *L = 2*.*85*, for KLF it is *38*.*73* at *L = 2*.*83*, for DLF it is *39*.*87* at *L = 2*.*84*, for PLF it is *38*.*91* at *L = 2*.*83*, and for the classical setup it is *40*.*61* at *L = 2*.*88* (cf. Table 4). These results conclude that the MSELF demonstrate the superior performance in detecting shifts in large sample sizes as compared to the other LFs.
6. The control chart based on the classical setup demonstrates inferior performance compared to the charts using the Bayesian setup. For example, with *δ = 1*.*2* and *n = 10*, the classical setup gives an *ARL*_1_ value of *72*.*28*, while the SELF gives an *ARL*_1_ value of *56*.*91* at *L = 2*.*96*, WSELF gives an *ARL*_1_ value of *55*.*62* at L = *2*.*95*, MSELF gives an *ARL*_*1*_ value of *53*.*38* at *L = 2*.*95*, KLF gives an *ARL*_1_ value of *55*.*47* at *L = 2*.*95*, DLF chart gives an *ARL*_1_ value of *57*.*79* at *L = 2*.*94* and PLF gives an *ARL*_1_ value of *57*.*20* at *L = 2*.*95*. (cf. Table 3). This indicates that the Bayesian setup can lead to better performance in control charts than the classical setup.
7. As the sample size increases from *10 to 15*, the magnitude of difference in *ARL*_1_ values for control chart under MSELF is superior to other charts SELF, WSELF, KLF, DLF and PLF. Specifically, with *δ = 1*.*2*, there are *32*.*74%*, *31*.*41%*, *32*.*78%*, *30*.*18%*, *31*.*01%* and *31*.*97%* decreases in *ARL*_1_ values for SELF, WSELF, MSELF, KLF, DLF and PLF respectively (cf. Tables 3 and 4). The results suggested that MSELF is superior choice for control charts when dealing with large sample sizes.
8. Additionally, it has been observed that the *ARL*_1_ at *λ* = 0.1 is smaller than *ARL*_1_ at *λ* = 0.2. It means that the efficiency of the designed control chart is improved at *λ* = 0.1 as compared to that at *λ* = 0.2 for all sample sizes (cf. 2–7). This implies that the efficiency of the proposed control chart is increased by decreasing the values of *λ*.

**Table 2:** ARL, SDRL and MDRL comparison using frequentist and Bayesian setups under different loss functions for n = 5, *λ* = 0.1 at ARL_0_ = 370.

δ	Classical			SELF			WSELF			MSELF			KLF			DLF			PLF		
	ARL	SDRL	MDRL	ARL	SDRL	MDRL	ARL	SDRL	MDRL	ARL	SDRL	MDRL	ARL	SDRL	MDRL	ARL	SDRL	MDRL	ARL	SDRL	MDRL
	L = 2.62			L = 3.13			L = 3.13			L = 3.13			L = 3.13			L = 3.13			L = 3.13		
1.2	212.97	207.39	150	90.76	74.78	69	91.46	78.81	68	88.29	75.99	66	91.11	77.13	68	93.01	77.67	71	91.8948	78.09	69
1.4	127.29	124.62	90	40.37	28.48	33	40.80	29.28	33	39.27	29.15	32	40.47	28.60	33	41.47	29.05	34	41.83	29.18	34
1.6	80.64	74.73	59	25.01	15.08	22	24.23	15.09	21	23.39	15.09	20	24.57	15.14	21	25.36	15.27	22	25.21	15.40	22
1.8	56.31	49.11	42	17.72	9.56	16	17.28	9.80	16	16.32	9.42	15	17.45	9.70	16	18.05	9.87	16	18.08	9.82	16
2	41.44	35.13	32	13.59	6.99	13	13.29	7.06	12	12.43	6.95	11	13.72	7.08	13	14.05	6.90	13	13.85	7.03	13
2.2	31.83	25.66	26	11.15	5.50	10	10.98	5.47	10	10.10	5.44	9	11.10	5.54	10	11.45	5.47	11	11.35	5.50	11
2.6	20.89	14.98	18	8.27	3.90	8	7.98	3.87	8	7.41	3.74	7	8.18	3.88	8	8.48	3.84	8	8.49	3.94	8
3	15.33	10.19	13	6.57	3.05	6	6.29	2.98	6	5.81	2.90	5	6.48	3.03	6	6.80	3.04	7	6.66	2.99	6

**Table 3:** ARL, SDRL and MDRL comparison using frequentist and Bayesian setups under different loss functions for n = 10, *λ* = 0.1 at ARL0 = 370.

δ	Classical			SELF			WSELF			MSELF			KLF			DLF			PLF		
	ARL	SDRL	MDRL	ARL	SDRL	MDRL	ARL	SDRL	MDRL	ARL	SDRL	MDRL	ARL	SDRL	MDRL	ARL	SDRL	MDRL	ARL	SDRL	MDRL
	L = 3.05			L = 2.96			L = 2.95			L = 2.95			L = 2.95			L = 2.94			L = 2.95		
1.2	72.28	61.25	54	56.91	41.94	45	55.63	42.06	44	53.39	41.26	42	55.48	40.79	44	57.80	42.05	46	57.21	42.35	45
1.4	28.13	19.15	24	24.01	12.91	21	22.91	12.92	20	21.65	13.05	19	23.55	13.01	21	24.30	12.74	22	24.19	12.99	21
1.6	16.38	9.56	15	14.71	6.60	14	13.94	6.58	13	13.01	6.72	12	14.32	6.63	13	15.43	6.71	14	15.01	6.61	14
1.8	11.25	5.97	10	10.72	4.42	10	10.00	4.34	9	9.11	4.27	9	10.36	4.34	10	11.15	4.44	11	10.93	4.38	10
2	8.51	4.31	8	8.49	3.28	8	7.85	3.26	8	7.06	3.18	7	8.13	3.28	8	8.81	3.32	8	8.64	3.27	8
2.2	6.89	3.37	7	6.95	2.67	7	6.53	2.66	6	5.79	2.55	6	6.72	2.63	7	7.32	2.67	7	7.13	2.68	7
2.6	4.98	2.34	5	5.22	1.95	5	4.79	1.88	5	4.24	1.82	4	4.99	1.91	5	5.45	1.94	5	5.36	1.95	5
3	3.91	1.78	4	4.18	1.56	4	3.85	1.49	4	3.41	1.43	3	4.03	1.52	4	4.40	1.59	4	4.31	1.55	4

**Table 4:** ARL, SDRL and MDRL comparison using frequentist and Bayesian setups under different loss functions for n = 15, *λ* = 0.1 at ARL_0_ = 370.

δ	Classical			SELF			WSELF			MSELF			KLF			DLF			PLF		
	ARL	SDRL	MDRL	ARL	SDRL	MDRL	ARL	SDRL	MDRL	ARL	SDRL	MDRL	ARL	SDRL	MDRL	ARL	SDRL	MDRL	ARL	SDRL	MDRL
	L = 2.88			L = 2.83			L = 2.85			L = 2.85			L = 2.83			L = 2.84			L = 2.83		
1.2	40.62	30.11	33	38.29	24.52	32	38.15	24.94	31	35.89	25.00	30	38.73	25.08	32	39.88	25.20	33	38.91	24.20	33
1.4	15.91	9.04	14	16.54	7.29	15	15.71	7.52	14	14.33	7.41	13	16.23	7.40	15	17.36	7.48	16	17.06	7.50	16
1.6	9.39	4.55	9	10.54	3.98	10	9.82	4.04	9	8.79	3.98	8	10.22	4.04	10	11.10	4.12	11	10.83	3.99	10
1.8	6.70	3.07	6	7.75	2.81	7	7.16	2.76	7	6.25	2.72	6	7.49	2.78	7	8.22	2.82	8	7.98	2.79	8
2	5.16	2.28	5	6.18	2.11	6	5.66	2.10	5	4.88	2.00	5	5.91	2.12	6	6.58	2.15	6	6.36	2.14	6
2.2	4.21	1.83	4	5.11	1.74	5	4.67	1.72	5	4.03	1.64	4	4.90	1.73	5	5.50	1.79	5	5.29	1.76	5
2.6	3.10	1.31	3	3.88	1.30	4	3.50	1.24	3	3.02	1.18	3	3.70	1.29	4	4.18	1.33	4	4.05	1.33	4
3	2.51	1.02	2	3.13	1.05	3	2.83	1.01	3	2.44	0.93	2	3.01	1.03	3	3.41	1.08	3	3.26	1.06	3

**Table 5:** ARL, SDRL and MDRL comparison using frequentist and Bayesian setups under different loss functions for n = 5, *λ* = 0.2 at ARL_0_ = 370.

	Classical			SELF			WSELF			MSELF			KLF			DLF			PLF		
δ	ARL	SDRL	MDRL	ARL	SDRL	MDRL	ARL	SDRL	MDRL	ARL	SDRL	MDRL	ARL	SDRL	MDRL	ARL	SDRL	MDRL	ARL	SDRL	MDRL
	L = 2.89			L = 3.99			L = 3.96			L = 3.96			L = 3.99			L = 3.96			L = 3.98		
1.2	229.93	233.26	158	114.07	108.68	81	110.8	105.74	78	112.3	108.7	78	115.26	110.47	82	113.92	109.12	81	114.54	108.99	81
1.4	154.41	151.85	108	51.52	45.9	38	50.28	45.82	37	50.36	46.09	36	51.76	47.15	38	51.74	45.81	38	51.7	46.11	38
1.6	109.93	107.77	77	29.6	24.69	23	28.42	23.91	22	28.57	24.2	22	28.96	24.19	22	29.27	24.12	22	29.36	24.28	23
1.8	78.89	76.77	56	18.72	14.6	15	18.8	14.69	15	18.27	14.61	14	19.01	14.84	15	19.09	14.94	15	19.2	14.96	15
2	59.32	57.18	42	13.91	10.35	11	13.58	9.87	11	13.29	10.15	11	13.93	10.21	11	13.94	9.93	11	13.99	10.1	12
2.2	45.32	43.19	33	10.65	7.16	9	10.58	7.25	9	10.29	7.26	9	10.74	7.4	9	10.89	7.27	9	10.87	7.24	9
2.6	29.96	27.5	22	7.5	4.61	7	7.25	4.53	6	7.06	4.6	6	7.38	4.64	6	7.58	4.65	7	7.5	4.62	7
3	20.89	18.16	16	5.74	3.31	5	5.51	3.31	5	5.34	3.31	5	5.66	3.36	5	5.81	3.36	5	5.78	3.32	5

**Table 6:** ARL, SDRL and MDRL comparison using frequentist and Bayesian setups under different loss functions for n = 10, *λ* = 0.2 at ARL_0_ = 370.

δ	Classical			SELF			WSELF			MSELF			KLF			DLF			PLF		
	ARL	SDRL	MDRL	ARL	SDRL	MDRL	ARL	SDRL	MDRL	ARL	SDRL	MDRL	ARL	SDRL	MDRL	ARL	SDRL	MDRL	ARL	SDRL	MDRL
	L = 3.85			L = 3.65			L = 3.65			L = 3.67			L = 3.65			L = 3.65			L = 3.65		
1.2	107.47	102.78	77	76.74	70.63	55	76.59	71.28	54	76.56	71.94	55	77.34	71.91	55	75.53	69.81	54	77.56	71.14	56
1.4	40.38	35.9	30	27.98	22.29	21	27.02	21.59	21	27.05	22.10	21	27.74	21.91	22	27.36	21.15	21	28.19	22.20	22
1.6	20.57	16.27	16	14.85	9.98	12	14.46	10.00	12	14.14	10.11	12	14.51	9.80	12	14.87	9.64	13	14.77	9.81	12
1.8	12.86	9.23	11	9.77	5.78	9	9.47	5.71	8	9.04	5.64	8	9.61	5.77	8	9.85	5.60	9	9.81	5.70	9
2	9.03	6.02	8	7.22	3.87	7	6.91	3.86	6	6.63	3.90	6	7.05	3.80	6	7.43	3.90	7	7.40	3.96	7
2.2	6.88	4.28	6	5.78	2.91	5	5.50	2.84	5	5.24	2.91	5	5.67	2.87	5	5.91	2.89	6	5.83	2.87	5
2.6	4.75	2.71	4	4.09	1.92	4	3.93	1.92	4	3.70	1.92	3	4.03	1.93	4	4.24	1.95	4	4.19	1.95	4
3	3.59	1.92	3	3.27	1.46	3	3.09	1.42	3	2.89	1.42	3	3.19	1.46	3	3.36	1.45	3	3.31	1.47	3

**Table 7:** ARL, SDRL and MDRL comparison using frequentist and Bayesian setups under different loss functions for n = 15, *λ* = 0.2 at ARL_0_ = 370.

δ	Classical			SELF			WSELF			MSELF			KLF			DLF			PLF		
	ARL	SDRL	MDRL	ARL	SDRL	MDRL	ARL	SDRL	MDRL	ARL	SDRL	MDRL	ARL	SDRL	MDRL	ARL	SDRL	MDRL	ARL	SDRL	MDRL
	L = 3.54			L = 3.44			L = 3.44			L = 3.45			L = 3.42			L = 3.43			L = 3.42		
1.2	56.72	51.89	41	49.83	43.37	37	48.01	41.15	36	48.46	42.53	36	47.62	41.69	35	49.52	41.61	37	49.27	42.08	37
1.4	18.68	14.5	15	16.73	11.37	14	15.91	11.08	13	15.63	11.25	13	16.11	11.02	13	16.79	10.87	14	16.97	11.28	14
1.6	9.78	6.46	8	9.25	5.08	8	8.86	5.12	8	8.37	4.97	7	8.99	5.00	8	9.48	5.09	9	9.44	5.08	8
1.8	6.32	3.71	6	6.31	3.05	6	6.01	3.08	6	5.64	3.07	5	6.12	3.02	6	6.58	3.13	6	6.47	3.09	6
2	4.7	2.62	4	4.82	2.15	5	4.58	2.21	4	4.23	2.17	4	4.72	2.22	4	5.03	2.22	5	4.95	2.21	5
2.2	3.76	1.94	3	3.91	1.7	4	3.72	1.69	4	3.40	1.67	3	3.78	1.69	4	4.11	1.72	4	4.04	1.72	4
2.6	2.7	1.31	3	2.92	1.2	3	2.70	1.16	3	2.47	1.14	2	2.79	1.19	3	3.03	1.20	3	2.96	1.20	3
3	2.14	0.99	2	2.34	0.93	2	2.16	0.91	2	1.99	0.86	2	2.25	0.92	2	2.45	0.96	2	2.38	0.94	2

Tables 2–7 and Figs 9–14 reveals that the Bayes estimates exhibit superior performance as compared to the classical estimates when it comes to ARL values for detecting shifts in the process for samples of size *n* = 5, 10 and 15. It is also revealed that EWMA control chart performs best for the Bayes estimates evaluated using MSELF as compares to those produced using the other LFs. Moreover, the performance of the chart improves further in detecting the shift by increasing the sample size. From Figs 9–14, it shows that the values of *ARL*_1_ decrease by increasing the shift. Moreover, the values of *ARL*_1_ under Bayesian approach for all the loss functions are less as compared to those for the classical case. It shows that Bayes estimates are more efficient in detecting the small shift than their classical counterparts.

## 7 Numerical study

To monitor the shape parameter *θ* of the IGD using Bayesian technique under different loss functions, the following algorithm will be followed:

1. Generate *k* samples each of size *n* = 5 from the *IGD* (1, 1).
2. Construct classical and Bayesian control limits for all the LFs under study.
3. Plot *Z*_*i*_, *i* = 1,2, …, *k* against the corresponding UCL for all the loss functions under study.
4. Declare the process to be out of control if *Z*_*i*_ falls beyond the respective UCL.

**Fig 15:**
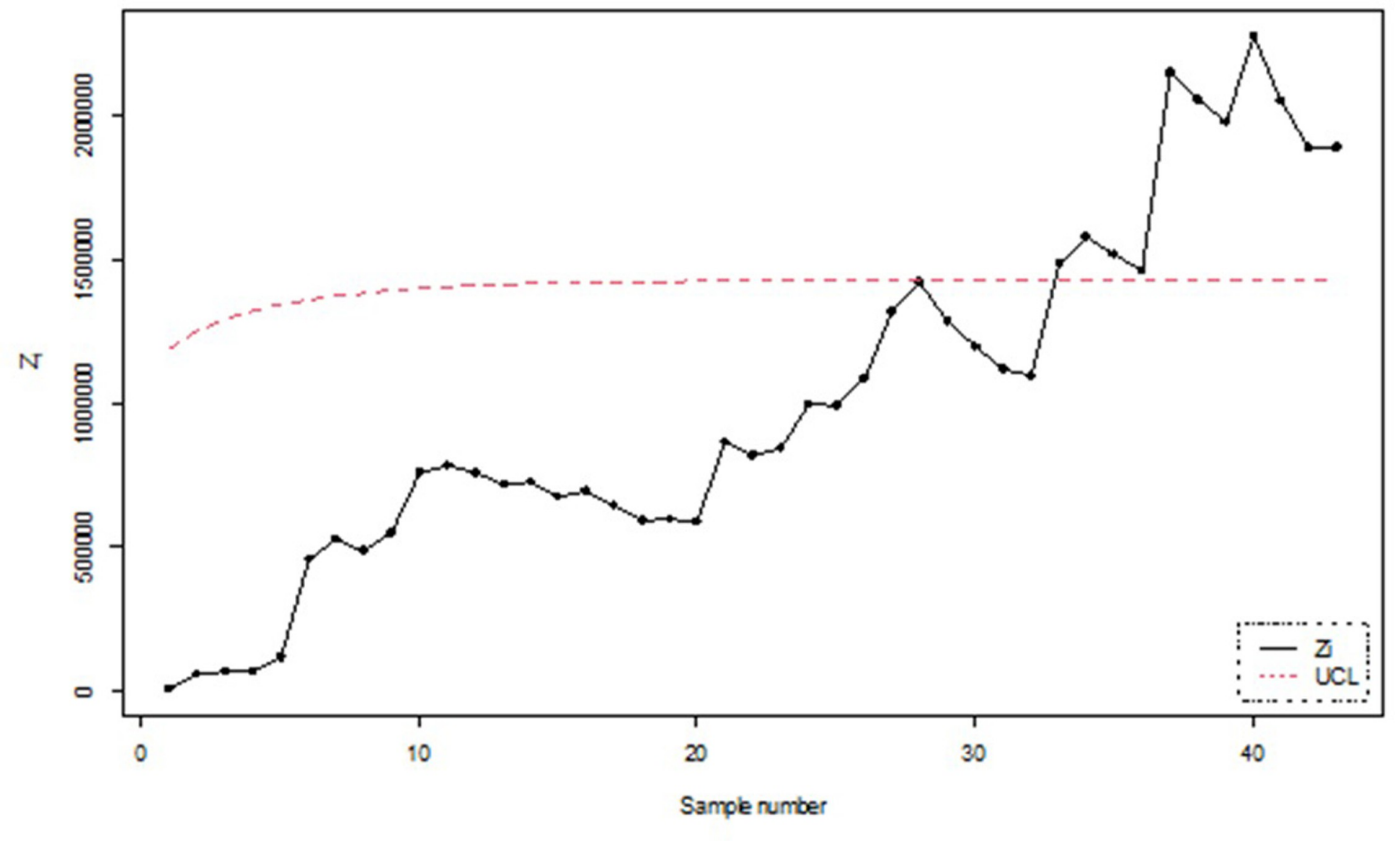
Classical process monitoring using simulated data for n = 5.

**Fig 16:**
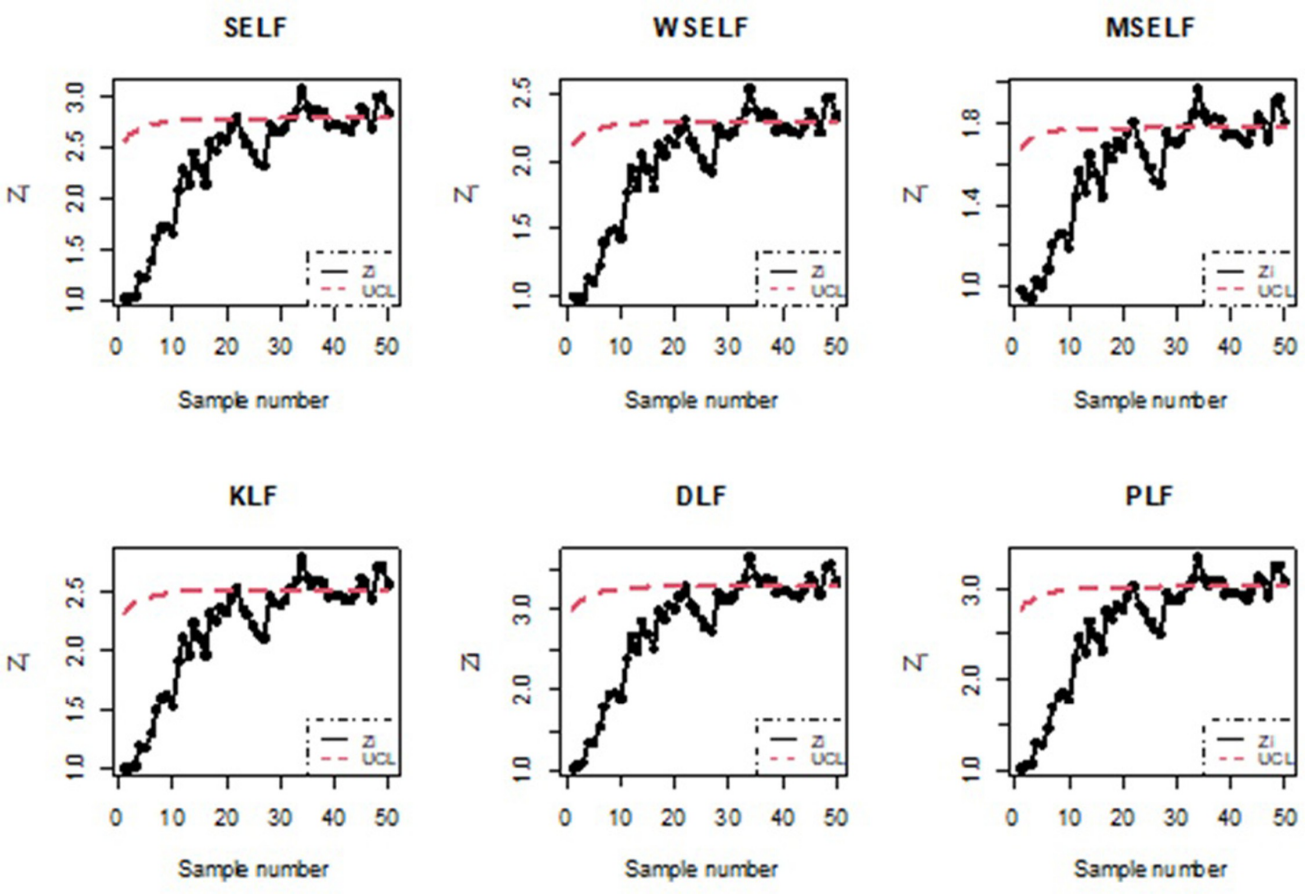
Bayesian process monitoring for all the loss functions using simulated data for n = 5.

To numerically illustrate the above-cited study, 50 samples each of size 5 were generated and the values of *Z*_*i*_, *i* = 1,2, …, 50 and the corresponding UCLs were computed. The graphical presentation of the resulting information is made in Figs 15 and 16.

Figs 15 and 16 illustrates the application of Bayesian process monitoring for the shape parameter of IGD across all the loss functions using the posterior distribution. The figure provides visual representation of sample number for which the EWMA statistic of *θ* falls outside the UCL and marked out-of-control alarm. The simulation results revealed that the Bayesian control chart with various loss functions resulted in a higher number of out-of-control alarms for the shape parameter as compared to classical control charts. Furthermore, the Bayesian approach outperforms the classical approach in detecting shifts in the process.

## 8 Real data application

This study illustrates how EWMA control chart can be employed for the detection of prospective changes in the IP of aerospace under frequentist and Bayesian approaches. This section demonstrates a practical application of the Bayesian control chart to monitor the shape parameter of IGD. To show the importance of the suggested methodology, its application is illustrated by real life data. Manufacturing data from the aerospace industry have been used for this purpose, (cf. industrial10.pdf (federalreserve.gov)). Khaliq et al. [67] also considered the data of aerospace industry from January 1997 to January 2015. As shown on this webpage, a brief description of the data/variables of interest has been provided related to the objectives of the study. Aerospace manufacturing includes the design, construction, testing, sale, and maintenance of airplanes, aircraft parts, missiles, rockets and spacecraft. There are several commercial and military applications for aerospace activity. This industry manufactures thousands of aircraft engine mounts, exhaust systems, landing gears and other components. There are numerous variables to consider. Industrial production (IP) index has been chosen as the quality characteristic of interest for this study. It evaluates the actual output of the manufacturing, mining, utility, and electric sectors. Data from January 1980 to December 2022 have been considered.

Prior to proceed with the Bayesian EWMA monitoring of the shape parameter of the IGD based on the real data, it is of prime importance to assess the goodness of fit of the model to the observed data. We may use the model selection criteria to evaluate the goodness of fit of the model to the observed data and compare different models, [68]. So we may use Akaike information criterion (AIC), [69] and Bayesian information criterion (BIC, also known as Schwarz criterion), which are respectively defined as

AIC=−2ln[L(μ,θ)]+2k

and

BIC=−2ln[L(μ,θ)]+kln(n)

Here *ln*[*L*(*μ, θ*)] denotes the log-likelihood, *n* denotes the number of observations and *k* denotes the number of parameters of the distribution under consideration. The smaller the values of these criteria are, the better the fit is. We have assumed the real-life data to follow the IGD and weibull distribution. So, we have evaluated the AIC and BIC for the data based on IGD and weibull model and their model selection criteria are reported in Table 8.

**Table 8:** Model selection criteria for IGD and weibull models.

Models	Estimates of		AIC	BIC
	*μ*	*θ*		
IGD	81.24159	1243.369	4552.227	4560.72
Weibull	2.865 × 10^10^	5.37	112012.8	112021.3

Here we have observed that the values of AIC and BIC for the IGD are very small as compared to those for the weibull distribution. Therefore, it can be concluded that the IGD gives a better fit to the real-life aerospace data and we can monitor the shape parameter of IGD based on the underlying real-life aerospace data.

**Fig 17:**
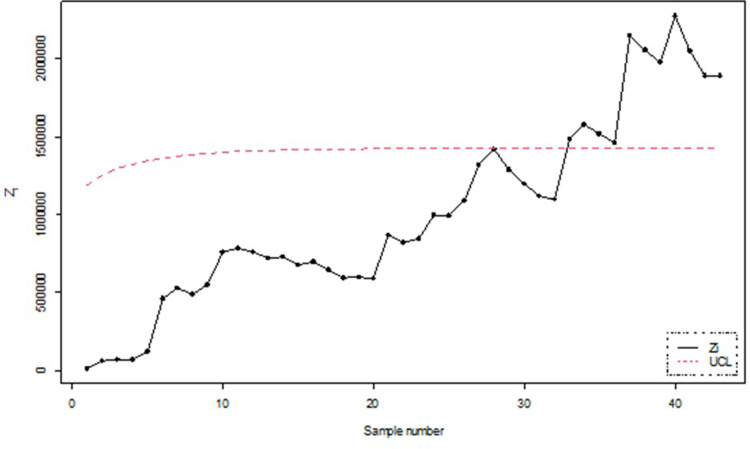
Classical process monitoring using the manufacturing data from the aerospace industry.

**Fig 18:**
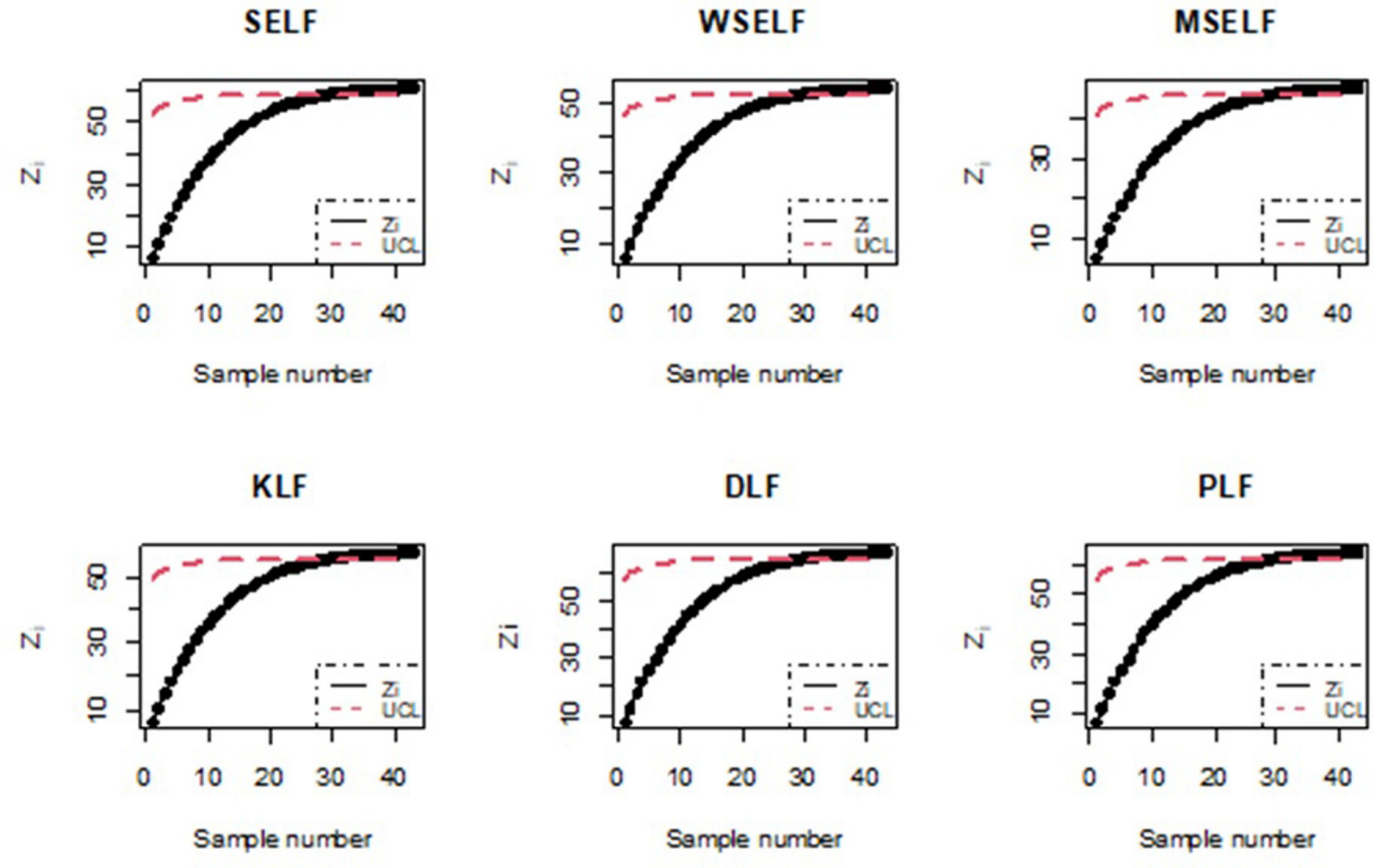
Bayesian process monitoring for all the loss functions using manufacturing data from the aerospace industry.

To numerically illustrate the above-cited study based on real data of 43 samples each of size 12, the values of *Z*_*i*_, *i* = 1,2, …, 43 and the corresponding UCLs were evaluated. The graphical presentation of the resulting information is made in Figs 17and 18.

Figs 17 and 18 illustrate the monitoring of the EWMA statistic of *θ* based on UCL computed for classical estimates and Bayes estimates obtained using the loss functions under study. Here we observed the similar pattern of shift detection by the control chart under study across all the estimation methods, i.e., the classical approach as well as the Bayesian approach for all the loss functions under study. Moreover, it is also noted that the Bayesian approach is more competent than classical approach in fault detection of the process.

## 9 Conclusion

The aim of this research is to design EWMA control charts under frequentist and Bayesian approaches to monitor the shape parameter of IGD. It is investigated that with increasing sample size, the recommended control charting method becomes increasingly effective in detecting process shifts. This study evaluates the impact of hyperparameter values on the Bayes estimates and posterior risks. Results showed that as hyperparameter *a* increases and *b* decreases, the values of BE increase across all loss functions. However, the values of PR decreases for SELF, WSELF, KLF and PLF. The simulation study reveals that the Bayes estimates are more efficient than classical estimates in detecting process shifts for all sample sizes. However, there is no significant difference in the performance of control chart for the loss functions under study. The study also reveals that the classical approach is unable to detect the out-of-control process, while the Bayesian approach is found to be more sensitive. It is further noted that the Bayesian EWMA control chart using MSELF outperforms all its competitors, and its performance is further improved by increasing sample size. The results of the entire study are based on the skewed waiting time distribution, namely the IGD. However, they may vary for other skewed distributions or symmetric distributions.

It is further recommended that the values of the hyperparameters may be elicited based on the experts’ opinion and the Bayes estimates may be evaluated using these values of the hyperparameters. We may extend this study for other competing distributions and competing charts.

## Supporting information

S1 File Real data. (CSV)

S2 File (DOCX)
