# Supplementary material for: Developing Bayesian EWMA chart for change detection in the shape parameter of Inverse Gaussian process
Source: PLoS One. 2024 May 6;19(5):e0301259. doi: 10.1371/journal.pone.0301259 (PMC11073744; doi:10.1371/journal.pone.0301259)
Supplement: S2 File — (DOCX) [file pone.0301259.s002.docx]

**Appendix A**

Suppose that $x_{1}, x_{2}, \ldots, x_{n}$ form a random sample of size $n$ from the IGD having the probability density function,

$f\left( x;\mu,\theta\right)=\left( \frac{\theta}{2\pi x^{3}} \right)^{1/2}\exp\left\{ -\frac{\theta\left( x-\mu\right)^{2}}{2\mu^{2}x} \right\}, x>0, \mu>0, \theta>0.$ (A.1)

The likelihood function is expressed as

$$p\left( x|\mu,\theta\right)=\prod_{i=1}^{n} f\left( x;\mu,\theta\right)=\prod_{i=1}^{n} \left[ \left( \frac{\theta}{2\pi{x_{i}}^{3}} \right)^{\frac{1}{2}}exp\left\{ -\frac{\theta\left( x_{i}-\mu\right)^{2}}{2\mu^{2}x_{i}} \right\} \right].$$

$=\left( \frac{\theta}{2\pi} \right)^{\frac{n}{2}}\prod_{i=1}^{n} \left( \frac{1}{x_{i}^{3}} \right)^{\frac{1}{2}}\exp\left\{ -\frac{\theta}{2\mu^{2}}\sum_{i=1}^{n} \frac{\left( x_{i}-\mu\right)^{2}}{x_{i}} \right\}.$ (A.2)

Let gamma distribution be the prior distribution for the shape parameter $\theta$ and expressed as:

$p\left( \theta\right)=\frac{b^{a}}{\Gamma(a)}\theta^{a-1}e^{-b\theta}, \theta>0, a>0,b>0.$ (A.3)

The posterior distribution of $\theta$ is:

$p(\theta|x) \propto p(\theta). p\left( x|\mu,\theta\right)$ (A.4)

$p\left( \theta| x \right)\propto\frac{b^{a}}{\Gamma\left( a \right)}\theta^{a-1}e^{-b\theta}. \left( \frac{\theta}{2\pi} \right)^{\frac{n}{2}}\prod_{i=1}^{n} \left( \frac{1}{x_{i}^{3}} \right)^{\frac{1}{2}}\exp\left\{ -\frac{\theta}{2\mu^{2}}\sum_{i=1}^{n} \frac{\left( x_{i}-\mu\right)^{2}}{x_{i}} \right\}.$

$p\left( \theta| x \right)\propto\left( \theta\right)^{\frac{n}{2}+a-1}. exp\left[ -\theta\left\{ b+\sum_{i=1}^{n} \frac{\left( x_{i}-\mu\right)^{2}}{{2\mu}^{2}x_{i}} \right\} \right].$ (A.5)

$\theta|x \sim Gamma\left( \frac{n}{2}+a,b+\sum_{i=1}^{n} \frac{\left( x_{i}-\mu\right)^{2}}{{2\mu}^{2}x_{i}} \right).$ (A.6)

where $\alpha=\frac{n}{2}+a$ and $\beta=b+\sum_{i=1}^{n} \frac{\left( x_{i}-\mu\right)^{2}}{{2\mu}^{2}x_{i}}.$

**Bayes estimator and posterior risk under DLF:** The BE under *DLF* is defined as:$\frac{E\left( \theta^{2}|\boldsymbol{x} \right)}{E\left( \theta|\boldsymbol{x} \right)}.$

$E\left( \theta| x \right)= \int_{0}^{\infty} \theta\frac{\beta^{\alpha}}{\Gamma\left( \alpha\right)}\theta^{\alpha-1}e^{-\beta\theta} d\theta$ (A.7)

$E\left( \theta| x \right)=\frac{\beta^{\alpha}}{\Gamma\left( \alpha\right)} \int_{0}^{\infty} \theta^{\alpha+1-1}e^{-\beta\theta} d\theta$

$E\left( \theta| x \right)=\frac{\beta^{\alpha}}{\Gamma\left( \alpha\right)} \frac{\Gamma(\alpha+1)}{\beta^{\alpha+1}} \Rightarrow E\left( \theta| x \right)= \frac{\Gamma(\alpha+1)}{\Gamma\left( \alpha\right) \beta}$

$E\left( \theta| x \right)=\frac{\alpha}{\beta}= \frac{\frac{n}{2}+a}{b+\sum_{i=1}^{n} \frac{\left( x_{i}-\mu\right)^{2}}{{2\mu}^{2}x_{i}}}$ (A.8)

and

$E\left( \theta^{2} | x \right)=\int_{0}^{\infty} \theta^{2}\frac{\beta^{\alpha}}{\Gamma\left( \alpha\right)}\theta^{\alpha-1}e^{-\beta\theta} d\theta$

$E\left( \theta^{2} | x \right)=\frac{\beta^{\alpha}}{\Gamma\left( \alpha\right)} \int_{0}^{\infty} \theta^{\alpha+1} e^{-\beta\theta} d\theta$

$E\left( \theta^{2} | x \right)=\frac{\beta^{\alpha}}{\Gamma\left( \alpha\right)} \frac{\Gamma\left( \alpha+2 \right)}{\beta^{\alpha+2}}$

$E\left( \theta^{2} | x \right)=\frac{\alpha(\alpha+1)}{\beta^{2}}=\frac{\left( \frac{n}{2}+a \right)\left( \frac{n}{2}+a+1 \right)}{\left[ b+\sum_{i=1}^{n} \frac{\left( x_{i}-\mu\right)^{2}}{{2\mu}^{2}x_{i}} \right]^{2}}$ (A.9)

So, Bayes estimator is:

$B.E=\frac{E\left( \theta^{2}|\boldsymbol{x} \right)}{E\left( \theta|\boldsymbol{x} \right)}$ (A.10)

$B.E\boldsymbol{=}\frac{\frac{\alpha\left( \alpha+1 \right)}{\beta^{2}}}{\frac{\alpha}{\beta}} \Rightarrow B.E\boldsymbol{=}\frac{\left( \alpha+1 \right)}{\beta}$

$B.E\boldsymbol{=}\frac{\left( \frac{n}{2}+a+1 \right)}{b+\sum_{i=1}^{n} \frac{\left( x_{i}-\mu\right)^{2}}{{2\mu}^{2}x_{i}}}$ (A.11)

The posterior risk under *DLF* is presented as:

$P.R=\frac{var\left( \theta|x \right)}{E\left( \theta^{2}|x \right)}$ (A.12)

$P.R=\frac{\frac{\alpha}{\beta^{2}}}{\frac{\alpha\left( \alpha+1 \right)}{\beta^{2}}} \Rightarrow P.R=\frac{1}{\left( \alpha+1 \right)} \Rightarrow P.R=\frac{1}{\left( \frac{n}{2}+a+1 \right)}$

$P.R=\frac{1}{\frac{n}{2}+a+1}$ (A.13)

**Appendix B**

**Notations/Abbreviations:**

| **Symbol / Abbreviation** | **Descriptions** | **Symbol / Abbreviation** | **Descriptions** |
| --- | --- | --- | --- |
| ARL | Average run length | RL | Run length |
| BE | Bayes estimator | SDRL | Standard deviation of run lengths |
| CUSUM | Cumulative sum | SELF | Squared-error loss function |
| PLF | Precautionary loss function | SPC | Statistical process control |
| DLF | DeGroot loss function | UCL | Upper control limit |
| EWMA | Exponentially weighted moving average | WSELF | Weighted- Squared error loss function |
| IGD | Inverse Gaussian distribution | ARL_0_ | In-control average run length |
| IP | Industrial production | ARL_1_ | Out-of-control average run length |
| KLF | K-loss function | *θ* | Shape parameter of IGD |
| LF | Loss function | *μ* | Location parameter |
| MRL | Mean deviation of run lengths | *δ* | Shift |
| MSELF | Modified Squared-error loss function | $\lambda$ | Smoothing constant |
| PR | Posterior risk | *a, b* | Hyperparameters |
